# No Trade-Off between Growth Rate and Temperature Stress Resistance in Four Insect Species

**DOI:** 10.1371/journal.pone.0062434

**Published:** 2013-04-30

**Authors:** Isabell Karl, Robby Stoks, Stephanie S. Bauerfeind, Anneke Dierks, Kristin Franke, Klaus Fischer

**Affiliations:** 1 Zoological Institute & Museum, University of Greifswald, Greifswald, Germany; 2 Laboratory of Aquatic Ecology, Evolution and Conservation, University of Leuven, Leuven, Belgium; Oxford Brookes University, United Kingdom

## Abstract

Although fast growth seems to be generally favored by natural selection, growth rates are rarely maximized in nature. Consequently, fast growth is predicted to carry costs resulting in intrinsic trade-offs. Disentangling such trade-offs is of great ecological importance in order to fully understand the prospects and limitations of growth rate variation. A recent study provided evidence for a hitherto unknown cost of fast growth, namely reduced cold stress resistance. Such relationships could be especially important under climate change. Against this background we here investigate the relationships between individual larval growth rate and adult heat as well as cold stress resistance, using eleven data sets from four different insect species (three butterfly species: *Bicyclus anynana, Lycaena tityrus*, *Pieris napi*; one Dipteran species: *Protophormia terraenovae*). Despite using different species (and partly different populations within species) and an array of experimental manipulations (e.g. different temperatures, photoperiods, feeding regimes, inbreeding levels), we were not able to provide any consistent evidence for trade-offs between fast growth and temperature stress resistance in these four insect species.

## Introduction

By definition life-history traits are closely related to fitness, and are consequently subject to trade-offs constraining their independent evolution [1]–[2]. Among the large number of life history traits, growth rate has recently received much attention, as it profoundly affects age and size at maturity and therefore adult fitness [3]. Although fast growth is typically favored by natural selection [4]–[5], growth rates are rarely maximized in nature [3]–[4], [6]. Instead, substantial genetic variation as well as plastic increases in growth rates are commonly found [6]–[9]. Hence, fast growth seems to be limited by intrinsic trade-offs [10]–[12], and lower than maximal growth rates result from an adaptive balancing of benefits and costs [5], [13].

The majority of studies on the costs of fast growth focus on ecological costs, such as a reduced locomotor and escape performance [10], [14]–[15], or a greater mortality through predation [12], [16]–[17]. Other studies targeted physiological costs of fast growth including an accumulation of damage in molecules, cells or tissues [18]–[20], reduced starvation resistance [5], [19], [21] and immune function [22]–[25]. Such patterns are thought to result from resource-allocation trade-offs, with an increased expenditure to fast growth reducing performance in other traits. The above studies further show that variation in larval growth rates regularly impact on fitness-related traits in the adult stage [26]–[27].

A recent study on damselflies provided evidence for a novel cost of rapid growth in terms of reduced cold stress resistance [28]. Consistent with the idea that rapid growth incurs energetic costs [29], populations exhibiting higher growth rates showed reduced expression of heat shock protein 70, supporting the hypothesis that faster growing individuals should perform worse at suboptimal temperatures [5]. Thus, growth patterns may directly influence adult temperature stress resistance. This finding has important implications as variation in environmental factors is thought to be the main source of variation in mortality [30]. In particular, temperature as a key environmental factor constitutes an important selective agent [31]–[32].

In order to test for the generality of the above pattern, we here use eleven data sets from four insect species (three butterflies, one Dipteran fly) to investigate trade-offs between individual growth rate and cold stress resistance. In extension to the study of Stoks and DeBlock [28], we test for phenotypic and genetic associations, and test for trade-offs between growth rate and heat stress resistance, thus testing for a general link between growth rate and performance under temperature stress. Our data sets include genetically divergent populations, some of which differ in growth rate, as well as various experimental manipulations, such as different thermal, photoperiodic and feeding regimes, allowing us to test for respective trade-offs in different environments and across populations.

## Materials and Methods

### Study species

We used eleven data sets from four different insect species to investigate the relationship between larval growth rates and temperature stress resistance, namely three Lepidopteran (*Bicyclus anynana* (Butler, 1879), *Lycaena tityrus* (Poda, 1761), *Pieris napi* (Linnaeus, 1758)) and one Dipteran species (*Protophormia terraenovae* (Robineau-Desvoidy, 1830)). Note that most data sets presented here have been used in previous publications already, addressing a wide array of questions such as inbreeding depression, selection on cold tolerance, local adaptation or plasticity in thermal resistance [33]–[41]. However, data are analyzed here in a completely new context which has not been addressed in any of the previous publications.

*Bicyclus anynana* (experiments 1 and 2) is a tropical, fruit-feeding butterfly, distributed from southern Africa to Ethiopia [42]. In 1988, a laboratory stock population was established at Leiden University, the Netherlands, from which a stock population was established at Greifswald University, Germany, in 2007 [37], [43]. *Lycaena tityrus* (experiments 3 to 7) is a widespread temperate-zone butterfly, ranging from Western Europe to central Asia [44]. All datasets on this species originate from F1 offspring of wild-caught females from populations near Greifswald (Northern Germany), in Bavaria (Southern Germany), or from mid-(1300–1500 m) or high-altitudes (1900–2100 m) of the Austrian and Italian Alps [33]–[35], [45]. *Pieris napi* (experiments 8 to 10) is a temperate zone butterfly that is widely distributed across the northern hemisphere [46]. All datasets on this species comprise F1 offspring of wild-caught females from populations near Greifswald (Northern Germany). *Protophormia terraenovae* (experiment 11) is a widespread temperate-zone fly with a holarctic distribution [47]. The flies used here originated from a laboratory stock population kept at Greifswald University for at least 200 generations. Flies were originally collected in the vicinity of Greifswald [36].

### Experimental design

Throughout, larval growth rates were determined as ln (natural logarithm) pupal mass/larval development time. Pupal mass was always weighed on day 1–2 after pupation. Temperature stress resistance was measured as chill-coma recovery time, i.e. the time needed to regain mobility after cold exposure, or as heat knock-down time, i.e. the time until physical knock-down under heat exposure. Both measures are considered reliable proxies of climatic cold and heat adaptation, respectively, and have been used successfully in the insects studied here by revealing expected patterns [33]–[36], [45], [48]. For measuring chill-coma recovery time, insects were individually placed in small translucent plastic cups (125 ml), which were arranged on trays in a randomized block design, and afterwards exposed to the cold (*B. anynana*: 19 h to 1°C; *L. tityrus*: 6 min to −20°C; *P. napi*: 19 h to −5°C; *P. terraenovae*: 20 h to −5°C). Heat knock-down times were measured at 45°C (*B. anynana, P. napi*) or 47°C (*L. tityrus*). Note that the patterns obtained are largely independent of the specific conditions used to measure temperature stress resistance [48]. Below we shortly describe the experiments from which the 11 data sets stem.

#### Experiments 1–2 (*Bicyclus anynana*)

All individuals used in *experiment 1* had been reared at a constant temperature of 27°C. Three different levels of inbreeding were established using a full-sib breeding design: inbreeding 1 (I1) with individuals resulting from matings between full sibs, inbreeding 2 (I2) resulting from matings between full sibs in two consecutive generations, and outbred controls (C) resulting from random mating (for details see [37]). Chill-coma recovery and heat knock-down time were measured and subsequently analyzed in relation to inbreeding level, sex and block (comprised of the individuals which were tested at a time).

*Experiment 2* involved 12 lines selected for increased cold tolerance and according controls (see [38]). First, three levels of inbreeding had been established as outlined above (I1, I2, C). Per inbreeding level, four lines were set up, two for selection on increased cold tolerance (CT), and two as unselected controls (UC). Per generation and line 40 males and 40 females were selected to found the next generation, being either the most cold-tolerant ones (CT) or being selected at random (UC). Selection was applied to chill-coma recovery time on day 1 following adult eclosion. Selection was continued for 10 generations, yielding highly divergent phenotypes with the lines selected for increased cold tolerance showing a by 28% shorter chill-coma recovery time compared to unselected controls [38]. Lines had been kept without selection for 4 generations prior to this experiment. The selection lines were randomly divided among two larval rearing temperatures (20 and 27°C, using 10 replicate cages each) and two adult acclimation temperatures (20 and 27°C, see [41]). While the butterflies reared at 27°C were a last time divided among two feeding treatments, being fed with banana (control) or water only (starvation), all animals reared at 20°C were fed with banana ad libitum. Chill-coma recovery time was measured at 20°C after 19 h exposure to 1°C, and analyzed in relation to (a) selection regime, replicate line, inbreeding level, rearing temperature, adult temperature, and sex, and in relation to (b) selection regime, replicate line, inbreeding level, adult temperature, adult feeding treatment, and sex (for individuals reared at 27°C).

#### Experiments 3–7 (*Lycaena tityrus*)

*Experiment 3* is based on butterflies caught near Bayreuth, southern Germany. Larvae were reared in full-sib families at two temperatures (20 and 27°C). Resulting butterflies were randomly divided among two adult acclimation temperatures (20 and 27°C), resulting in 4 treatment groups [45]. Chill-coma recovery time was measured and analyzed in relation to rearing temperature, adult temperature, family, and sex. In *experiment 4* offspring from females caught in the vicinity of Greifswald, northeast Germany, were used. Larvae were reared at two mean temperatures (18 and 24°C) under constant or fluctuating thermal conditions, thus resulting in 4 treatment groups [35]. Chill-coma recovery and heat knock-down time were measured and analyzed in relation to mean temperature, temperature variation (constant versus fluctuating), and sex.

Individuals for *experiment 5* originate from replicated low- (500–600 mNN) and high-altitude (1900–2100 mNN) populations, being reared at 18°C or 27°C [33]. Chill-coma recovery and heat knock-down time were measured and analyzed in relation to altitude, replicate population, rearing temperature, and sex. In *experiment 6* butterflies from replicated low-(500–600 mNN), mid-(1300–1500 mNN) and high-altitude (1900–2100 m NN) populations were reared at 27°C [33]. Chill-coma recovery and heat knock-down time were measured and analyzed in relation to altitude, replicate population, and sex.

The butterflies used in *experiment 7* originated from low-altitude populations (near Greifswald, Westerburg and Benediktbeuern; all Germany) and were either reared at 19°C or 24°C. After determination of chill-coma recovery and heat knock-down time, butterflies were killed and phosphoglucose isomerase (PGI) genotypes were identified by gel electrophoresis. Subsequent analyses involved PGI genotypes PGI 1–1, PGI 2–2, PGI 1–2, and PGI 2–3 [34]. Chill-coma recovery and heat knock-down time were measured and analyzed in relation to genotype, rearing temperature, and sex.

#### Experiments 8–10 (*Pieris napi*)

In *experiment 8* offspring from females caught near Greifswald were reared at four different thermal regimes, differing in temperature mean and amplitude: (1) mean temperature: 17°C, amplitude 7°C; (2) mean temperature: 20°C, amplitude: 7°C; (3) mean temperature: 20°C, amplitude: 12°C; (4) mean temperature of 17°C and amplitude of 7°C during the first half of larval development, and mean temperature of 27°C and amplitude of 7°C during the second half of larval development. Chill-coma recovery and heat knock-down time were measured and analyzed in relation to temperature regime and sex. For *experiments 9 and 10* also females caught near Greifswald were used, with offspring being reared at 20°C or 27°C (*experiment 9*) and at 19°C or 25°C (*experiment 10*). In both experiments, chill-coma recovery and heat knock-down time were measured and analyzed in relation to rearing temperature (block, in *experiment 9* only) and sex.

#### Experiment 11 (*Protophormia terraenovae*)

In experiment 11 stock flies were reared at two temperatures (20 and 27°C) and at two photoperiods (12 h and 18 h light), resulting in four treatments groups. Chill-coma recovery time was measured and analyzed in relation to rearing temperature, photoperiod, and sex.

### Statistical analyses

To test for associations between larval growth rate and cold-or heat stress resistance we used linear mixed models with growth rate added as continuous variable for all experiments. For the factors used in the respective models please see under experimental design above. Throughout, replicate lines or populations were nested within the respective higher order factor (for details see Tables S1, S2, S3, S4). Replicate, family and block were included as random effects, whilst all other factors were considered fixed effects. Throughout we computed full-factorial models including all interactions terms between categorical factors. These models were used to test whether growth rate has a significant impact on stress tolerance. Overall slopes (SL)±SE for growth rate and temperature stress resistance traits were provided for all data sets based on the above mentioned analyses.

In an additional set of mixed models we tested for interactions between the variable growth rate and full factors (see Tables S5, S6, S7, S8), which we have not considered in the above analyses. These analyses tested whether slopes were homogeneous across treatment groups. In case of significant interactions involving growth rate, slopes were computed separately for groups with a homogeneous slope. Furthermore, we calculated Pearson's product moment correlations between temperature stress resistance traits and growth rate within all individual treatment groups involved in our experiments (n = 206; see Tables S9, S10, S11, S12). Note that a positive correlation (or slope) between growth rate and chill-coma recovery time reflects a negative association between fast growth and cold stress resistance (and thus a trade-off, as faster growing individuals need longer to recover), while a positive correlation between growth rate and heat knock-down time reflects a positive association between fast growth and heat stress resistance (faster growing individuals resist heat stress for longer). All statistical tests were performed using Statistica 8 (StatSoft, Tulsa, OK, USA).

Note that below we only highlight associations between growth rate and chill coma recovery and heat knock-down time, respectively, while discarding other effects which have been analyzed and discussed in detail elsewhere (see above and [33]–[41]).

## Results

**Figure 1:**
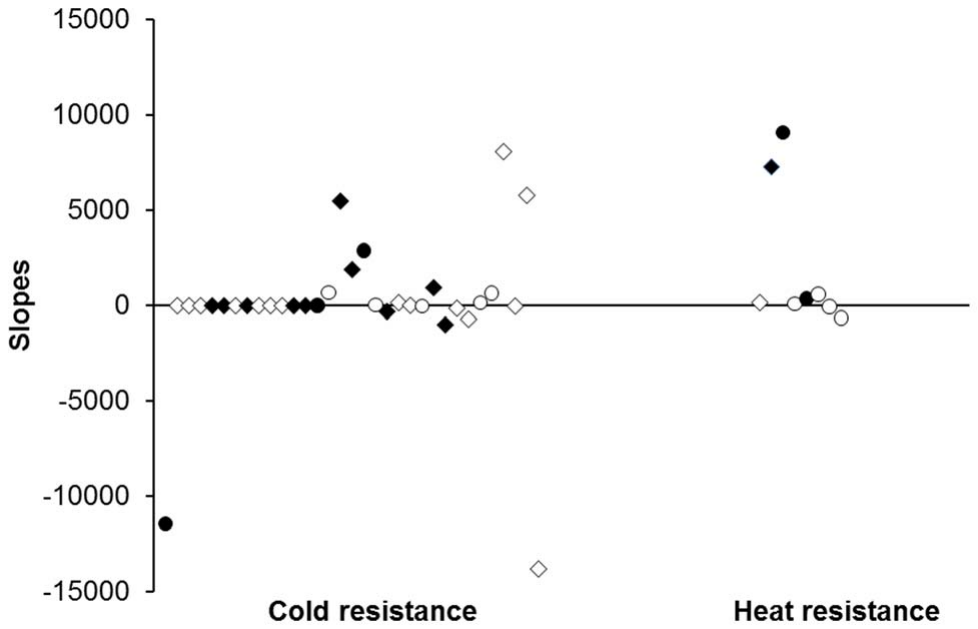
Slopes for the associations between insect larval growth rates and temperature stress resistance (left: chill-coma recovery time; right: heat knock-down time). Data are based on eleven experiments using four different insect species. Given are overall slopes, i.e. across all treatment groups within an experiment (circles), and slopes for subgroups with homogeneous slopes (diamonds). Filled symbols: significant slopes; open symbols: non-significant slopes. Slopes are from left to right arranged in the order of experiments, thus starting with experiment 1. Note that trade-offs between growth rate and stress resistance are indicated by positive slopes for cold resistance, but by negative slopes for resistance.

Our linear (mixed) model analyses using 11 experiments on in total four insect species revealed that growth rate added as continuous variable had a significant impact on chill-coma recovery time in 3 out of 12 cases, and on heat knock-down time in 2 out of 8 cases (Tables S1, S2, S3, S4). Additionally including interactions between growth rate and categorical factors revealed a very similar pattern, showing in only 2 out of 12 (chill-coma recovery time) and 2 out of 8 (heat knock-down time) cases a significant effect of growth rate (Tables S5, S6, S7, S8). The slopes across all treatment groups for growth rate and stress resistance traits were one time positive (indicating a trade-off), two times negative and 9 times non-significant for chill-coma recovery time, and two times positive and 6 times non-significant for heat knock-down time (Table 1, Fig. 1). Slopes across subgroups of homogeneous slopes were 5 times positive (indicating a trade-off), 5 times negative and 15 times non-significant for chill-coma recovery time, and one time positive and one time negative (indicating a trade-off) for heat knock-down time. Finally, within-group correlations with growth rate were only in 20 out of 146 cases significant for chill-coma recovery time, being 10 times positive (indicating a trade-off) and 10 times negative, and only in 10 out of 60 cases for heat knock-down time, being 6 times positive and 4 times negative (indicating a trade-off; Tables S9, S10, S11, S12).

**Table 1:** Overview over the associations found between insect larval growth rates and temperature stress resistance traits (chill-coma recovery and heat knock-down time).

	Positive	Negative	N.S.
**Slopes across treatment groups**			
Chill coma recovery time	1	2	9
Heat knock-down time	2	0	6
**Slopes across subgroups**			
Chill coma recovery time	5	5	15
Heat knock-down time	1	1	0
**Correlations within treatment groups**			
Chill coma recovery time	10	10	126
Heat knock-down time	6	4	50

Given are the numbers of significantly positive, significantly negative, and non-significant (N.S.) slopes/correlations for (a) overall slopes across all treatment groups within the respective analysis, for (b) the slopes of subgroups with homogeneous slopes, and for (c) Pearson correlations within each individual treatment group. Note that trade-offs between growth rate and stress resistance are indicated by positive slopes/correlations for chill coma recovery time, but negative slopes/correlations for heat knock-down time.

## Discussion

Recent studies suggest that growth rate is a life-history trait in its own rights, which can be optimized by natural selection [3]–[4], [6]. The fact that growth rates are often not maximized strongly implies that fast growth carries costs [3]–[4], [6], [49]. Thus, growth rate is predicted to be involved in trade-offs. It has been hypothesized that such trade-offs may result in faster growing individuals performing poorer at suboptimal temperatures [5]. This prediction has potentially large implications, as temperature is a key environmental factors for ectotherms strongly affecting survival [32], [50]–[51]. Current climate change is likely to pose additional stress on many species, once again underscoring the importance of considering such trade-offs [52]–[54].

In a recent study Stoks and De Block [28] provided empirical support for a trade-off between growth rate and cold resistance, by demonstrating reduced cold stress resistance in fast growing individuals and populations of the damselfly *Ischnura elegans*. Consequently, the reduced cold resistance of southern populations may not only result from relaxed thermal selection, but also from the costs of higher growth rates selected for by a change in voltinism [28]. However, such patterns may be complicated by differences in ambient temperature and genetic backgrounds across populations. We have therefore tested here for the generality of the above pattern, focusing on resource allocation trade-offs between growth rate and temperature stress resistance in different populations of four insect species and across several environmental conditions. Our results though revealed no support for a (general) relationship between growth rate and either cold or heat stress resistance, despite using large sample sizes with concomitantly high statistical power, and different experimental settings. This result was not affected by the statistical approach used, as neither mixed models nor standard correlations revealed any meaningful support. Most results regarding the impact of growth rate were non-significant, suggesting that growth rate is in general not tightly linked to temperature stress resistance in the four studied insect species.

Obviously, traits related to fitness may show wide variation within and across species driven by adaptive evolution [55]–[57], which may substantially affect expected trade-offs. The four insect species used in our study are distributed from southern Africa through to (sub-) polar regions. However, the lack of support for a trade-off between growth rate and temperature stress resistance prevailed across species, populations, and environmental conditions. For *L. tityrus*, only 1 out of 4 mixed model analyses revealed a significant impact of growth rate on cold tolerance (6 significant out of 46 within-group correlations), and only 2 out of 4 times a significant impact of growth rate on heat tolerance (7 significant out of 36 within-group correlations). In *P. napi* and *P. terranovae* the variable growth rate was non-significant throughout, and only 4 out of 40 correlations were significant. In the tropical butterfly *B. anynana* though all 2 mixed model analyses showed a significant impact of growth rate on cold tolerance, but not on heat tolerance. However, slopes as well as correlations did not reveal any consistent pattern, being sometimes positive, sometimes negative (see also Table 1). Generally, life history trade-offs are notoriously difficult to detect under benign (feeding) conditions and across genetically divergent populations [58]–[60]. However, given the large number of treatments used here, including groups exposed to food stress as well as genetically divergent populations, such complications can hardly account for our negative results.

In conclusion, we have found no evidence for a general trade-off between temperature stress resistance and growth rate in the four studied insect species. Most tests were non-significant, and the significant ones revealed inconsistent patterns. Given the use of various species and populations, being tested under an array of environmental conditions, we argue that such a general trade-off does not exist in insects, even though this notion rests on a small number of species only. While for the time being rejecting a general pattern, our findings, of course, do not rule out the existence of trade-offs between growth rate and temperature stress resistance under specific conditions or some other insect orders (as suggested for Odonata [28]).

## Supporting Information

Table S1 Experiments 1 and 2 (*Bicyclus anynana*). In *experiment 1*, linear mixed models revealed a significant effect of growth rate on chill-coma recovery but not on heat knock-down time (Table S1a). The overall slope was negative for chill-coma recovery time (SL = −11450±3650, N = 384). The lack of significant interactions between growth rate and other factors suggests that slopes were homogeneous across treatment groups for chill-coma recovery time, while for heat knock-down time the sex by growth rate interaction was significant (Table S5). The resulting slopes for subgroups of homogeneous slopes were non-significant for males (SL = 160±1800, P = 0.929, N = 134) but significant for females (SL = 7300±1800, P<0.001, N = 155). Within treatment groups, 2 out of 6 correlations with growth rates were significant for both, chill-coma recovery time and heat knock-down time (Table S9). For the former trait, both significant correlations were negative, while they were positive for the latter trait. In *experiment 2*, linear mixed models revealed no significant impact of growth rate on chill-coma recovery time for *experiment 2A*, but for *experiment 2B* (Table S1a; *experiment 2B*: SL = −0.4±0.2, N = 3091). Mixed models including interactions with the variable growth rate revealed 5 and 8 significant interactions for *experiments 2A* and *2B*, respectively (Table S5), suggesting wide variation in slopes across treatment groups. The slopes for the resulting groups of homogeneous slopes in *experiment 2a* were 3× negative, 2× positive, and 7× non-significant (Table S1b). For *experiment 2B* growth rate significantly interacted with all other factors (Table S5), such that no slopes for sub-groups can be given. Within-group correlations (for both *experiments 2A* and *2B*) revealed only 9 significant out of 70 correlations between growth rate and chill-coma recovery time, 4 positive and 5 negative ones (Table S9). **Table S1a**: Results of linear (mixed) models for (1) the effects of inbreeding level (Inbreeding), sex, and block (random factor) on chill-coma recovery (CCR) and heat knock-down time (HKD) for the butterfly *Bicyclus anynana* (*experiment 1*); for (2) selection regime (Selection), replicate line (nested within selection regime, random), inbreeding level (Inbreeding), rearing temperature (RT), acclimation temperature (AT), and sex on chill-coma recovery time (*experiment 2A*); and for (3) the effects of selection regime (Selection), replicate line (nested within selection regime, random), inbreeding level (Inbreeding), acclimation temperature (AT), adult feeding treatment (Food), and sex on chill-coma recovery time (*experiment 2B*). Growth rate (GR) was included as continuous variable throughout. Significant p-values are given in bold. **Table S1b**: Slopes for growth rate versus chill-coma recovery time for the groups with homogeneous slopes for *experiment 2A* (i.e. across inbreeding levels (Inbreed), rearing temperatures (RT), and acclimation temperatures (AT)). Significant p-values are given in bold.
(DOCX)

Table S2 Experiments 3–7 (*Lycaena tityrus*). Growth rate added as continuous variable did not significantly affect chill-coma recovery time in *experiment 3* (Table S2). There were furthermore no significant interactions between growth rate and other factors (Table S6). Concomitantly, only 1 out of 8 within-group correlations was significant, showing a positive correlation between growth rate and chill-coma recovery time (Table S10). In *experiment 4* the variable growth rate significantly affected both chill-coma recovery and heat knock-down time (Table S2). The overall slopes across treatment groups revealed a positive relation between growth rate and both chill-coma recovery time (SL = 2900±840, N = 383) and heat knock-down time (SL = 9100±3300, N = 376). The lack of significant interactions between growth rate and other factors suggests that slopes were homogeneous across treatment groups for heat knock-down time, but not for chill-coma recovery time (significant GR x Sex interaction, Table S6). The resulting slopes for subgroups of homogeneous slopes were significant for males (SL = 5500±2400, P = 0.021, N = 155) and females (SL = 1900±400, P<0.001, N = 226). Regarding correlations within treatment groups, 3 (all positive) out of 8 correlations between growth rate and chill-coma recovery time were significant, while only 1 (negative) out of 8 correlations between growth rate and heat knock-down time was significant (Table S10). In *experiment 5* the variable growth rate neither significantly affected chill-coma recovery nor heat knock-down time (Table S2). Further, for chill-coma recovery time as well as for heat knock-down time no interactions between growth rate and other factors were significant (Table S6). Within-group correlations were significant in 1 out of 8 cases for both, chill-coma recovery and heat-knock-down time, being both times negative (Table S10). In *experiment 6* linear mixed models again revealed no significant effect of the continuous variable growth rate on chill-coma recovery or heat knock-down time (Table S2). However, including interactions with the growth rate revealed significant differences in slopes among treatment groups (Table S6). For chill-coma recovery time, the interaction between altitude and growth rate was significant. However, only the slope for the low-altitude populations was significant (being negative: SL = −300±130, P = 0.027, N = 103), but not the ones for mid- (SL = 160±120, P = 0.176, N = 84) and high-altitude populations (SL = 20±70, P = 0.797, N = 94). For heat-knock-down time growth rate interacted significantly with all other factors, such that no slopes for subgroups could be calculated. Within-group correlations with growth rate were in 1 out of 6 cases significant for chill-coma recovery time, and in 2 out of 6 cases for heat knock-down time (Table S10). In the former case the significant correlation was negative, while in the latter case 1 positive and 1 negative correlation was found. In *experiment 7* growth rate significantly affected heat knock-down (SL = 360±70, N = 654), but not chill-coma recovery time (Table S2). While including interactions with the continuous variable growth rate revealed a homogeneous slope for chill-coma recovery time, a significant genotype by sex by growth rate interaction was found for heat knock-down time (Table S6). Thus no slopes for subgroups could be calculated. Within-group correlations with growth rate were in all 16 cases non-significant for chill-coma recovery time, and in only 3 (all positive) out of 16 cases significant for heat knock-down time (Table S10). **Table S2**: Results of linear (mixed) models for (1) the effects of rearing temperature (RT), acclimation temperature (AT), family (random factor), and sex on chill-coma recovery (CCR) in the butterfly *Lycaena tityrus* (*experiment 3*); for (2) the effects of mean temperature (Temperature), temperature variation (Variation), and sex on chill-coma recovery and heat knock-down time (HKD) (*experiment 4*); for (3) the effects of altitude (Altitude), replicate population (Repl., nested within altitude, random), rearing temperature (RT), and sex on chill-coma recovery and heat knock-down time (*experiment 5*); for (4) the effects of altitude (Altitude), replicate population (nested within altitude, random), and sex on chill-coma recovery and heat knock-down time (*experiment 6*); and (5) for the effects of genotype, rearing temperature (RT), and sex on chill-coma recovery and heat knock-down time (*experiment 7*). Growth rate (GR) was included as continuous variable throughout. Significant p-values are given in bold.
(DOCX)

Table S3 Experiments 8–10 (*Pieris napi*). In *experiment 8* growth rate had neither a significant impact on chill-coma recovery nor on heat knock-down time (Table S3). Including interactions with growth rate revealed a significant thermal regime by growth rate interaction for chill-coma-recovery time, while slopes were homogeneous for heat knock-down time (Table S7). The resulting slopes for subgroups were significant for chill-coma recovery time in groups 1 (SL = 950±470, P = 0.048, N = 76) and 2 (−990±480, P = 0.041, N = 68), but not in groups 3 (−130±500, P = 0.799, N = 77) and 4 (−700±700, P = 0.328, N = 36). Regarding within-group correlations with growth rate, only 1 out of 8 correlations was significant for chill-coma recovery time (being positive), while none was significant for heat knock-down time (Table S11). In *experiment 9* neither chill-coma recovery nor heat knock-down time was significantly affected by growth rate (Table S3). The lack of interactions between growth rate and other factors further suggests that slopes are homogeneous across treatment groups (Table S7). Within-group correlations showed in 1 out of 4 cases a significantly negative correlation between growth rate and heat-knock-down time, while no correlation was significant for chill-coma recovery time (Table S11). In *experiment 10* linear models did not reveal a significant effect of growth rate on chill-coma recovery or heat knock-down time (Table S3). Slopes were homogeneous across treatment groups as indicated by the lack of significant interactions with the variable growth rate (Table S8). Regarding within-group correlations, 1 (negative) out of 4 correlations with growth rate were significant for chill-coma recovery time, while none was significant for heat knock-down time (Table S11). **Table S3**: Results of linear (mixed) models for (1) the effects of thermal regime (TR) and sexon chill-coma recovery (CCR) and heat knock-down time (HKD) in *Pieris napi* (*experiment 8*); for (2) rearing temperature (RT), block and sex on chill-coma recovery and heat knock-down time (*experiment 9*) and on rearing temperature (RT) and sex on chill-coma recovery and heat knock-down time (*experiment 10*). Growth rate (GR) was included as continuous variable throughout. Significant p-values are given in bold.
(DOCX)

Table S4 Experiment 11 (*Protophormia terraenovae*). In *experiment 11*, finally, growth rate added as continuous variable showed no significant impact on chill-coma recovery time (Table S4). However, slopes were not homogeneous across treatment groups, as indicated by a significant photoperiod by growth rate and a rearing temperature by photoperiod by growth rate interaction (Table S8). The resulting slopes were non-significant in all four cases (20°C/12 h: 8100±5900, P = 0.179, N = 46; 20°C/18 h: −240±4600, P = 0.958, N = 46; 27°C/12 h: 5800±5400, P = 0.288, N = 47; 27°C/18 h: −13800±7900, P = 0.086, N = 46;). Only 1 out of 8 within-group correlations was significant, showing a positive correlation between growth rate and chill-coma recovery time (Table S12). **Table S4**: Results of a linear model for the effects of rearing temperature (RT), photoperiod (PhP), and sex on chill-coma recovery (CCR) in *Protophormia terranovae*. Growth rate (GR) was included as continuous variable. Significant p-values are given in bold.
(DOCX)

Table S5 Experiments 1 and 2 (*Bicyclus anynana*). Results of linear (mixed) models including interactions with the continuous variable growth rate (GR) for the butterfly *Bicyclus anynana* used in experiments 1 and 2. In *experiment 1*, effects of inbreeding level (Inbreeding), sex and block (random factor) on chill-coma recovery (CCR) and heat knock-down time (HKD) were investigated. In *experiment 2* two separate analyses were used (see Methods). First, the effects of selection regime (Selection), replicate line (nested within selection regime, random), inbreeding level (Inbreeding), rearing temperature (RT), acclimation temperature (AT), and sex (*experiment 2A*), and second the effects of selection regime (Selection), replicate line (nested within selection regime, random), inbreeding level (Inbreeding), acclimation temperature (AT), adult feeding treatment (Food), and sex on chill-coma recovery time were investigated (*experiment 2B*). Growth rate (GR) was included as continuous variable throughout. Significant p-values are given in bold.
(DOCX)

Table S6 Experiments 3–7 (*Lycaena tityrus*). Results of linear (mixed) models including interactions with the continuous variable growth rate (GR) for the butterfly *Lycaena tityrus* used in *experiments 3*–*7*. In *experiment 3* the effects of rearing temperature (RT), acclimation temperature (AT), family (random factor), and sex on chill-coma recovery (CCR) were investigated; in *experiment 4* the effects of mean temperature (Temperature), temperature variation (Variation), and sex on chill-coma recovery and heat knock-down time (HKD), respectively, were investigated; in *experiment 5* the effects of altitude, replicate population (nested within altitude, random), rearing temperature (RT), and sex on chill-coma recovery and heat knock-down time, respectively, were investigated; in *experiment 6* the effects of altitude, replicate population (nested within altitude, random), and sex on chill-coma recovery and heat knock-down time, respectively, were investigated; in *experiment 7* the effect of PGI genotype, rearing temperature (RT), and sex on chill-coma recovery and heat knock-down time, respectively, were investigated. Growth rate (GR) was included as continuous variable throughout. Significant p-values are given in bold.
(DOCX)

Table S7 Experiments 8–10 (*Pieris napi*). Results of linear models including interactions with the continuous variable growth rate (GR) for the butterfly *Pieris napi* used in *experiments 8*–*10*. In *experiment 8* the effects of thermal regime (TR) and sex on chill-coma recovery (CCR) and heat knock-down time (HKD), respectively, were investigated; in *experiment 9* the effects of rearing temperature (RT), block and sex on chill-coma recovery and heat knock-down time and in *experiment 10* the effects of rearing temperature (RT) and sex on chill-coma recovery and heat knock-down time, respectively, were investigated. Growth rate (GR) was included as continuous variable throughout. Significant p-values are given in bold.
(DOCX)

Table S8 Experiment 11 (*Protophormia terraenovae*). Results of an linear model including interactions with the continuous variable growth rate (GR) for the fly *Protophormia terranovae* used in *experiment 11*. The effects of rearing temperature (RT), photoperiod (PhP), sex, and the continuous variable growth rate on chill-coma recovery (CCR) were investigated. Significant p-values are given in bold.
(DOCX)

Table S9 Experiments 1 and 2 (*Bicyclus anynana*). Within-group correlations between growth rate and temperature stress resistance (chill coma recovery, CCR and/or heat knock down time, HKD) for the butterfly *Bicyclus anynana* in *experiment 1* (N = 6 correlations per trait) and *experiment 2* (N = 70 correlations). Inbreeding (Inb) 1 = inbreeding level 1, Inbreeding (Inb) 2 = inbreeding level 2, Inbreeding (Inb) 0 = outbred controls; Sel 0 = unselected control lines, Sel 1 = cold selected lines; RT = rearing temperature (20 or 27°C); AT = acclimation temperature (20 or 27°C); F 0 = starvation, F 1 = no starvation; M = male, F = female. Significant correlations are given in bold.
(DOCX)

Table S10 Experiments 3–7 (*Lycaena tityrus*). Within-group correlations between growth rate and temperature stress resistance (chill coma recovery, CCR and/or heat knock down time, HKD) for the butterfly *Lycaena tityrus* in *experiments 3*–*7* (N = 8–16 correlations per trait). RT = rearing temperature (18, 19, 20, 24 or 27°C); AT = acclimation temperature (20 or 27°C); const. = const rearing conditions, fluct. = fluctuating rearing conditions; low = low-altitude populations (0–600 m), mid = mid-altitude populations (1300–1500 m), high = high-altitude populations (1900–2100 m); G1 = PGI 1–1, G2 = PGI 2–2, G3 = PGI 1–2, G4 = PGI 2–3 genotypes; M = male, F = female. Significant correlations are given in bold.
(DOCX)

Table S11 Experiments 8–10 (*Pieris napi*). Within-group correlations between growth rate and temperature stress resistance (chill-coma recovery, CCR and/or heat knock-down time, HKD) for the butterfly *Pieris napi* in *experiments 8*–*10* (N = 4–8 correlations per trait). TR1 = mean temperature: 17°C, amplitude 7°C; TR2 = mean temperature: 20°C, amplitude: 7°C; TR3 = mean temperature: 20°C, amplitude: 12°C; TR 4 = mean temperature of 17°C and amplitude of 7°C during the first half of larval development and mean temperature of 27°C and amplitude of 7°C during the second half of larval development; RT = rearing temperature (19, 20, 25 or 27°C); M = male, F = female. Significant correlations are given in bold.
(DOCX)

Table S12 Experiment 11 (*Protophormia terraenovae*). Within-group correlations between growth rate and chill-coma recovery (CCR) for the fly *Protophormia terranovae* in *experiment 11* (N = 8 correlations). RT = rearing temperature (20 or 27°C); 12 h/18 h = photoperiod of 12 or 18 h; M = male, F = female. Significant correlations are given in bold.
(DOCX)
