# Supplementary material for: No Trade-Off between Growth Rate and Temperature Stress Resistance in Four Insect Species
Source: PLoS One. 2013 Apr 30;8(4):e62434. doi: 10.1371/journal.pone.0062434 (PMC3640073; doi:10.1371/journal.pone.0062434)
Supplement: Table S1 — Experiments 1 and 2 (Bicyclus anynana). In experiment 1, linear mixed models revealed a significant effect of growth rate on chill-coma recovery but not on heat knock-down time (Table S1a). The overall slope was negative for chill-coma recovery time (SL = −11450±3650, N = 384). The lack of significant interactions between growth rate and other factors suggests that slopes were homogeneous across treatment groups for chill-coma recovery time, while for heat knock-down time the sex by growth rate interaction was significant (Table S5). The resulting slopes for subgroups of homogeneous slopes were non-significant for males (SL = 160±1800, P = 0.929, N = 134) but significant for females (SL = 7300±1800, P<0.001, N = 155). Within treatment groups, 2 out of 6 correlations with growth rates were significant for both, chill-coma recovery time and heat knock-down time (Table S9). For the former trait, both significant correlations were negative, while they were positive for the latter trait. In experiment 2, linear mixed models revealed no significant impact of growth rate on chill-coma recovery time for experiment 2A, but for experiment 2B (Table S1a; experiment 2B: SL = −0.4±0.2, N = 3091). Mixed models including interactions with the variable growth rate revealed 5 and 8 significant interactions for experiments 2A and 2B, respectively (Table S5), suggesting wide variation in slopes across treatment groups. The slopes for the resulting groups of homogeneous slopes in experiment 2a were 3× negative, 2× positive, and 7× non-significant (Table S1b). For experiment 2B growth rate significantly interacted with all other factors (Table S5), such that no slopes for sub-groups can be given. Within-group correlations (for both experiments 2A and 2B) revealed only 9 significant out of 70 correlations between growth rate and chill-coma recovery time, 4 positive and 5 negative ones (Table S9). Table S1a: Results of linear (mixed) models for (1) the effects of inbreeding level (Inbreed [file pone.0062434.s001.docx]

**Table S1a**

|  |  |  |  |  |  |
| --- | --- | --- | --- | --- | --- |
| **Experiment 1** | **Source** | **MS** | **DF** | **F** | **P** |
| CCR | Inbreeding | 927393 | 2 | 1.10 | 0.333 |
|  | Sex | 1414963 | 1 | 1.68 | 0.195 |
|  | Block | 1406459 | 28 | 1.67 | **0.019** |
|  | Inbreeding*Sex | 379737 | 2 | 0.45 | 0.636 |
|  | GR | 8278588 | 1 | 9.84 | **0.001** |
|  | Error | 840683 | 349 |  |  |
| HKD | Inbreeding | 132344 | 2 | 0.51 | 0.602 |
|  | Sex | 1038065 | 1 | 3.98 | **0.047** |
|  | Block | 4095632 | 23 | 15.71 | **< 0.001** |
|  | Inbreeding*Sex | 112997 | 2 | 0.43 | 0.648 |
|  | GR | 487317 | 1 | 1.87 | 0.172 |
|  | Error | 260670 | 258 |  |  |
| **Experiment 2 (A)** | **Source** | **MS** | **DF** | **F** | **P** |
| CCR | Selection | 19000.51 | 1 | 22.49 | **0.042** |
|  | Repl.[Sel] | 842.79 | 2 | 6.14 | **0.002** |
|  | Inbreeding | 546.96 | 2 | 3.98 | **0.019** |
|  | RT | 7122.47 | 1 | 51.94 | **<0.001** |
|  | AT | 89625.38 | 1 | 653.66 | **<0.001** |
|  | Sex | 25.05 | 1 | 0.18 | 0.669 |
|  | Sel.*Inbreed. | 82.47 | 2 | 0.60 | 0.548 |
|  | Sel.*RT | 516.90 | 1 | 3.76 | 0.052 |
|  | Inbreed.*RT | 393.96 | 2 | 2.87 | 0.057 |
|  | Sel.*AT | 929.45 | 1 | 6.77 | **0.009** |
|  | Inbreed.*AT | 564.88 | 2 | 4.11 | **0.016** |
|  | RT*AT | 88.06 | 1 | 0.64 | 0.423 |
|  | Sel.*Sex | 10.60 | 1 | 0.07 | 0.781 |
|  | Inbreed.*Sex | 761.61 | 2 | 5.55 | **0.004** |
|  | RT*Sex | 38.42 | 1 | 0.28 | 0.597 |
|  | AT*Sex | 2300.55 | 1 | 16.77 | **<0.001** |
|  | Sel.*Inbreed.*RT | 587.72 | 2 | 4.28 | **0.014** |
|  | Sel.*Inbreed.*AT | 431.63 | 2 | 3.14 | **0.043** |
|  | Sel.*RT*AT | 563.53 | 1 | 4.11 | **0.043** |
|  | Inbreed.*RT*AT | 362.76 | 2 | 2.64 | **0.071** |
|  | Sel.*Inbreed.*Sex | 35.33 | 2 | 0.25 | 0.773 |
|  | Sel.*RT*Sex | 2.00 | 1 | 0.01 | 0.904 |
|  | Inbreed.*RT*Sex | 329.99 | 2 | 2.40 | 0.090 |
|  | Sel.*AT*Sex | 162.39 | 1 | 1.18 | 0.277 |
|  | Inbreed.*AT*Sex | 425.80 | 2 | 3.10 | **0.045** |
|  | RT*AT*Sex | 2233.01 | 1 | 16.28 | **<0.001** |
|  | Sel.*Inbreed.*RT*AT | 524.69 | 2 | 3.82 | **0.022** |
|  | Sel.*Inbreed.*RT*Sex | 157.87 | 2 | 1.15 | 0.316 |
|  | Sel.*Inbreed.*AT*Sex | 18.61 | 2 | 0.13 | 0.873 |
|  | Sel.*RT*AT*Sex | 139.80 | 1 | 1.01 | 0.313 |
|  | Inbreed.*RT*AT*Sex | 24.56 | 2 | 0.17 | 0.836 |
|  | Sel.*Inbreed.*RT*AT*Sex | 70.17 | 2 | 0.51 | 0.600 |
|  | GR | 127.03 | 1 | 0.92 | 0.336 |
|  | Error | 137.11 | 3191 |  |  |
| **Experiment 2 (B)** | **Source** | **MS** | **DF** | **F** | **P** |
| CCR | Selection | 23117.12 | 1 | 7.83 | 0.108 |
|  | Repl.[Sel] | 2911.93 | 2 | 15.14 | **< 0.001** |
|  | Inbreeding | 2458.03 | 2 | 12.78 | **< 0.001** |
|  | AT | 82216.74 | 1 | 427.70 | **< 0.001** |
|  | Food | 0.25 | 1 | < 0.00 | 0.971 |
|  | Sex | 61.56 | 1 | 0.32 | 0.572 |
|  | Sel.*Inbreed. | 1029.92 | 2 | 5.35 | **0.005** |
|  | Sel.*AT | 358.37 | 1 | 1.86 | 0.172 |
|  | Inbreed.*AT | 1659.06 | 2 | 8.63 | **< 0.001** |
|  | Sel.*Food | 20.52 | 1 | 0.10 | 0.744 |
|  | Inbreed.*Food | 179.82 | 2 | 0.93 | 0.393 |
|  | AT*Food | 325.11 | 1 | 1.69 | 0.194 |
|  | Sel.*Sex | 71.13 | 1 | 0.37 | 0.543 |
|  | Inbreed.*Sex | 152.25 | 2 | 0.79 | 0.453 |
|  | AT*Sex | 3434.42 | 1 | 17.86 | **< 0.001** |
|  | Food*Sex | 212.20 | 1 | 1.10 | 0.293 |
|  | Sel.*Inbreed.*AT | 976.25 | 2 | 5.07 | **0.006** |
|  | Sel.*Inbreed.*Food | 54.06 | 2 | 0.28 | 0.755 |
|  | Sel.*AT*Food | 103.68 | 1 | 0.53 | 0.463 |
|  | Inbreed.*AT*Food | 143.38 | 2 | 0.74 | 0.474 |
|  | Sel.*Inbreed.*Sex | 54.38 | 2 | 0.28 | 0.754 |
|  | Sel.*AT*Sex | 73.40 | 1 | 0.38 | 0.537 |
|  | Inbreed.*AT*Sex | 98.37 | 2 | 0.51 | 0.599 |
|  | Sel.*Food*Sex | 142.31 | 1 | 0.74 | 0.390 |
|  | Inbreed.*Food*Sex | 1238.90 | 2 | 6.44 | **0.002** |
|  | AT*Food*Sex | 1113.74 | 1 | 5.79 | **0.016** |
|  | Sel.*Inbreed.*AT*Food | 215.50 | 2 | 1.12 | 0.326 |
|  | Sel.*Inbreed.*AT*Sex | 99.93 | 2 | 0.51 | 0.595 |
|  | Sel.*Inbreed.*Food*Sex | 385.25 | 2 | 2.00 | 0.135 |
|  | Sel.*AT*Food*Sex | 51.27 | 1 | 0.26 | 0.606 |
|  | Inbreed.*AT*Food*Sex | 208.13 | 2 | 1.08 | 0.339 |
|  | Sel.*Inbreed.*AT*Food*Sex | 158.36 | 2 | 0.82 | 0.439 |
|  | GR | 1496.10 | 1 | 7.78 | **0.005** |
|  | Error | 192.23 | 3040 |  |  |

**Table S1b**

| **Subgroups** | **SL ± SE** | **P** | **N** |
| --- | --- | --- | --- |
| Inbreed 0, RT 20, AT 20 | 0.8 ± 0.6 | 0.154 | 288 |
| Inbreed 0, RT 20, AT 27 | -0.3 ± 1.0 | 0.730 | 282 |
| Inbreed 0, RT 27, AT 20 | 0.5 ± 0.5 | 0.326 | 266 |
| Inbreed 0, RT 27, AT 27 | -1.5 ± 0.6 | **0.021** | 283 |
| Inbreed 1, RT 20, AT 20 | 2.8 ± 0.7 | **< 0.001** | 295 |
| Inbreed 1, RT 20, AT 27 | -0.7 ± 1.2 | 0.553 | 291 |
| Inbreed 1, RT 27, AT 20 | -1.5 ± 0.6 | **0.016** | 257 |
| Inbreed 1, RT 27, AT 27 | -0.5 ± 0.7 | 0.511 | 257 |
| Inbreed 2, RT 20, AT 20 | 1.0 ± 0.6 | 0.083 | 263 |
| Inbreed 2, RT 20, AT 27 | -0.8 ± 0.9 | 0.403 | 262 |
| Inbreed 2, RT 27, AT 20 | -2.0 ± 0.6 | **0.002** | 242 |
| Inbreed 2, RT 27, AT 27 | 1.9 ± 0.7 | **0.004** | 256 |
