# Supplementary material for: No Trade-Off between Growth Rate and Temperature Stress Resistance in Four Insect Species
Source: PLoS One. 2013 Apr 30;8(4):e62434. doi: 10.1371/journal.pone.0062434 (PMC3640073; doi:10.1371/journal.pone.0062434)
Supplement: Table S2 — Experiments 3–7 (Lycaena tityrus). Growth rate added as continuous variable did not significantly affect chill-coma recovery time in experiment 3 (Table S2). There were furthermore no significant interactions between growth rate and other factors (Table S6). Concomitantly, only 1 out of 8 within-group correlations was significant, showing a positive correlation between growth rate and chill-coma recovery time (Table S10). In experiment 4 the variable growth rate significantly affected both chill-coma recovery and heat knock-down time (Table S2). The overall slopes across treatment groups revealed a positive relation between growth rate and both chill-coma recovery time (SL = 2900±840, N = 383) and heat knock-down time (SL = 9100±3300, N = 376). The lack of significant interactions between growth rate and other factors suggests that slopes were homogeneous across treatment groups for heat knock-down time, but not for chill-coma recovery time (significant GR x Sex interaction, Table S6). The resulting slopes for subgroups of homogeneous slopes were significant for males (SL = 5500±2400, P = 0.021, N = 155) and females (SL = 1900±400, P<0.001, N = 226). Regarding correlations within treatment groups, 3 (all positive) out of 8 correlations between growth rate and chill-coma recovery time were significant, while only 1 (negative) out of 8 correlations between growth rate and heat knock-down time was significant (Table S10). In experiment 5 the variable growth rate neither significantly affected chill-coma recovery nor heat knock-down time (Table S2). Further, for chill-coma recovery time as well as for heat knock-down time no interactions between growth rate and other factors were significant (Table S6). Within-group correlations were significant in 1 out of 8 cases for both, chill-coma recovery and heat-knock-down time, being both times negative (Table S10). In experiment 6 linear mixed models again revealed no significant effect of the continuous variable growth rate o [file pone.0062434.s002.docx]

**Table S2**

|  |  |  |  |  |  |
| --- | --- | --- | --- | --- | --- |
| **Experiment 3** | **Source** | **MS** | **DF** | **F** | **P** |
| CCR | RT | 292023 | 1 | 0.73 | 0.391 |
|  | AT | 162562 | 1 | 0.41 | 0.522 |
|  | Family | 1090838 | 4 | 2.75 | **0.028** |
|  | Sex | 111952 | 1 | 0.28 | 0.595 |
|  | RT*AT | 718064 | 1 | 1.81 | 0.179 |
|  | RT*Sex | 623887 | 1 | 1.57 | 0.210 |
|  | AT*Sex | 794218 | 1 | 2.00 | 0.158 |
|  | RT*AT*Sex | 109 | 1 | < 0.00 | 0.987 |
|  | GR | 106916 | 1 | 0.27 | 0.604 |
|  | Error | 395899 | 329 |  |  |
| **Experiment 4** | **Source** | **MS** | **DF** | **F** | **P** |
| CCR | Temperature | 751694.7 | 1 | 9.64 | **0.002** |
|  | Variation | 30858.1 | 1 | 0.39 | 0.530 |
|  | Sex | 500524.8 | 1 | 6.42 | **0.012** |
|  | Temp.*Var. | 252682.9 | 1 | 3.24 | 0.073 |
|  | Temp.*Sex | 2291.5 | 1 | 0.02 | 0.864 |
|  | Var.*Sex | 433496.9 | 1 | 5.56 | **0.019** |
|  | Temp.*Var.*Sex | 5223.7 | 1 | 0.06 | 0.796 |
|  | GR | 951303.2 | 1 | 12.20 | **0.001** |
|  | Error | 77919.2 | 368 |  |  |
| HKD | Temperature | 26446907 | 1 | 23.31 | **< 0.001** |
|  | Variation | 25926500 | 1 | 22.85 | **< 0.001** |
|  | Sex | 2850640 | 1 | 2.51 | 0.114 |
|  | Temp.*Var. | 18642 | 1 | 0.01 | 0.898 |
|  | Temp.*Sex | 1151635 | 1 | 1.01 | 0.314 |
|  | Var.*Sex | 95653 | 1 | 0.08 | 0.772 |
|  | Temp.*Var.*Sex | 1148042 | 1 | 1.01 | 0.315 |
|  | GR | 8848686 | 1 | 7.79 | **0.006** |
|  | Error | 1134540 | 361 |  |  |
| **Experiment 5** | **Source** | **MS** | **DF** | **F** | **P** |
| CCR | Altitude | 674394.7 | 1,2 | 48.46 | **< 0.001** |
|  | Repl.[Alt.] | 1612.7 | 2,394 | 0.03 | 0.965 |
|  | Sex | 12434.0 | 1,394 | 0.27 | 0.602 |
|  | RT | 97606.9 | 1,394 | 2.14 | 0.144 |
|  | Alt.*Sex | 3710.6 | 1,394 | 0.08 | 0.776 |
|  | Alt.*RT | 54839.8 | 1,394 | 1.20 | 0.273 |
|  | Sex*RT | 16309.6 | 1,394 | 0.35 | 0.550 |
|  | Alt.*Sex*RT | 9009.0 | 1,394 | 0.19 | 0.657 |
|  | GR | 17913.5 | 1,394 | 0.39 | 0.531 |
|  | Fehler | 45570.8 | 394 |  |  |
| HKD | Altitude | 650565.8 | 1,3 | 13.63 | **< 0.001** |
|  | Repl.[Alt.] | 57337.5 | 2,390 | 1.20 | 0.302 |
|  | Sex | 241067.0 | 1,390 | 5.05 | **0.025** |
|  | RT | 93244.4 | 1,390 | 1.95 | 0.163 |
|  | Alt.*Sex | 36122.2 | 1,390 | 0.75 | 0.385 |
|  | Alt.*RT | 601429.7 | 1,390 | 12.60 | **< 0.001** |
|  | Sex*RT | 14.8 | 1,390 | < 0.00 | 0.986 |
|  | Alt.*Sex*RT | 15561.9 | 1,390 | 0.32 | 0.568 |
|  | GR | 134017.6 | 1,390 | 2.80 | 0.095 |
|  | Fehler | 47716.5 | 390 |  |  |
| **Experiment 6** | **Source** | **MS** | **DF** | **F** | **P** |
| CCR | Altitude | 1846049 | 2,3 | 17.46 | **0.022** |
|  | Repl. [Alt.] | 106974 | 3,271 | 4.08 | **0.007** |
|  | Sex | 15336 | 1,271 | 0.58 | 0.445 |
|  | Alt.*Sex | 6273 | 2,271 | 0.23 | 0.787 |
|  | GR | 47044 | 1,271 | 1.79 | 0.181 |
|  | Error | 26180 | 271 |  |  |
| HKD | Altitude | 3672868 | 2,3 | 287.05 | **< 0.001** |
|  | Repl. [Alt.] | 10885 | 3,270 | 0.14 | 0.933 |
|  | Sex | 1438338 | 1,271 | 19.03 | **< 0.001** |
|  | Alt.*Sex | 136853 | 2,271 | 1.81 | 0.165 |
|  | GR | 36913 | 1,271 | 0.48 | 0.485 |
|  | Error | 75577 | 271 |  |  |
| **Experiment 7** | **Source** | **MS** | **DF** | **F** | **P** |
| CCR | Genotype | 299557.7 | 3 | 4.02 | **0.007** |
|  | RT | 103834.0 | 1 | 1.39 | 0.238 |
|  | Sex | 172461.0 | 1 | 2.31 | 0.128 |
|  | Genotype*Temp. | 54533.7 | 3 | 0.73 | 0.533 |
|  | Genotype*Sex | 13772.7 | 3 | 0.18 | 0.907 |
|  | Temp.*Sex | 110894.5 | 1 | 1.49 | 0.223 |
|  | Genotype*Temp.*Sex | 53522.9 | 3 | 0.71 | 0.541 |
|  | GR | 1585.4 | 1 | 0.02 | 0.884 |
|  | Error | 74412.0 | 633 |  |  |
| HKD | Genotype | 19703 | 3 | 0.45 | 0.711 |
|  | RT | 404768 | 1 | 9.42 | **0.002** |
|  | Sex | 4183187 | 1 | 97.40 | **< 0.001** |
|  | Genotype*Temp. | 27204 | 3 | 0.63 | 0.594 |
|  | Genotype*Sex | 17352 | 3 | 0.40 | 0.750 |
|  | Temp.*Sex | 185547 | 1 | 4.32 | **0.038** |
|  | Genotype*Temp.*Sex | 9426 | 3 | 0.21 | 0.883 |
|  | GR | 1120194 | 1 | 26.08 | **< 0.001** |
|  | Error | 42944 | 637 |  |  |
