# Supplementary material for: No Trade-Off between Growth Rate and Temperature Stress Resistance in Four Insect Species
Source: PLoS One. 2013 Apr 30;8(4):e62434. doi: 10.1371/journal.pone.0062434 (PMC3640073; doi:10.1371/journal.pone.0062434)
Supplement: Table S3 — Experiments 8–10 (Pieris napi). In experiment 8 growth rate had neither a significant impact on chill-coma recovery nor on heat knock-down time (Table S3). Including interactions with growth rate revealed a significant thermal regime by growth rate interaction for chill-coma-recovery time, while slopes were homogeneous for heat knock-down time (Table S7). The resulting slopes for subgroups were significant for chill-coma recovery time in groups 1 (SL = 950±470, P = 0.048, N = 76) and 2 (−990±480, P = 0.041, N = 68), but not in groups 3 (−130±500, P = 0.799, N = 77) and 4 (−700±700, P = 0.328, N = 36). Regarding within-group correlations with growth rate, only 1 out of 8 correlations was significant for chill-coma recovery time (being positive), while none was significant for heat knock-down time (Table S11). In experiment 9 neither chill-coma recovery nor heat knock-down time was significantly affected by growth rate (Table S3). The lack of interactions between growth rate and other factors further suggests that slopes are homogeneous across treatment groups (Table S7). Within-group correlations showed in 1 out of 4 cases a significantly negative correlation between growth rate and heat-knock-down time, while no correlation was significant for chill-coma recovery time (Table S11). In experiment 10 linear models did not reveal a significant effect of growth rate on chill-coma recovery or heat knock-down time (Table S3). Slopes were homogeneous across treatment groups as indicated by the lack of significant interactions with the variable growth rate (Table S8). Regarding within-group correlations, 1 (negative) out of 4 correlations with growth rate were significant for chill-coma recovery time, while none was significant for heat knock-down time (Table S11). Table S3: Results of linear (mixed) models for (1) the effects of thermal regime (TR) and sexon chill-coma recovery (CCR) and heat knock-down time (HKD) in Pieris napi (experiment 8); for (2) rearing temperature ( [file pone.0062434.s003.docx]

**Table S3**

|  |  |  |  |  |  |
| --- | --- | --- | --- | --- | --- |
| **Experiment 8** | **Source** | **MS** | **DF** | **F** | **P** |
| CCR | TR | 316761 | 3 | 13.08 | **< 0.001** |
|  | Sex | 30651 | 1 | 1.26 | 0.262 |
|  | TR*Sex | 6563 | 3 | 0.27 | 0.846 |
|  | GR | 405 | 1 | 0.01 | 0.897 |
|  | Error | 24208 | 247 |  |  |
| HKD | TR | 148056 | 3 | 0.57 | 0.631 |
|  | Sex | 187824 | 1 | 0.73 | 0.393 |
|  | TR*Sex | 255302 | 3 | 0.99 | 0.396 |
|  | GR | 112234 | 1 | 0.43 | 0.509 |
|  | Error | 256855 | 222 |  |  |
| **Experiment 9** | **Source** | **MS** | **DF** | **F** | **P** |
| CCR | RT | 17139.0 | 1 | 0.11 | 0.731 |
|  | Sex | 96935.6 | 1 | 0.66 | 0.414 |
|  | Block | 370659.3 | 26 | 2.55 | **< 0.001** |
|  | RT*Sex | 248749.3 | 1 | 1.71 | 0.192 |
|  | GR | 5835.4 | 1 | 0.04 | 0.841 |
|  | Error | 144844.0 | 168 |  |  |
| HKD | RT | 602249 | 1 | 10.57 | **0.001** |
|  | Sex | 19454 | 1 | 0.34 | 0.560 |
|  | Block | 131792 | 22 | 2.31 | **0.002** |
|  | RT*Sex | 684 | 1 | 0.01 | 0.913 |
|  | GR | 50337 | 1 | 0.88 | 0.349 |
|  | Error | 56963 | 137 |  |  |
| **Experiment 10** | **Source** | **MS** | **DF** | **F** | **P** |
| CCR | RT | 237587 | 1 | 1.91 | 0.167 |
|  | Sex | 212482 | 1 | 1.71 | 0.191 |
|  | RT*Sex | 12785 | 1 | 0.10 | 0.748 |
|  | GR | 72288 | 1 | 0.58 | 0.445 |
|  | Error | 123878 | 356 |  |  |
| HKD | RT | 972141 | 1 | 14.12 | **< 0.001** |
|  | Sex | 156884 | 1 | 2.27 | 0.132 |
|  | RT*Sex | 17364 | 1 | 0.25 | 0.616 |
|  | GR | 86903 | 1 | 1.26 | 0.262 |
|  | Error | 68832 | 346 |  |  |
