# Supplementary material for: No Trade-Off between Growth Rate and Temperature Stress Resistance in Four Insect Species
Source: PLoS One. 2013 Apr 30;8(4):e62434. doi: 10.1371/journal.pone.0062434 (PMC3640073; doi:10.1371/journal.pone.0062434)
Supplement: Table S4 — Experiment 11 (Protophormia terraenovae). In experiment 11, finally, growth rate added as continuous variable showed no significant impact on chill-coma recovery time (Table S4). However, slopes were not homogeneous across treatment groups, as indicated by a significant photoperiod by growth rate and a rearing temperature by photoperiod by growth rate interaction (Table S8). The resulting slopes were non-significant in all four cases (20°C/12 h: 8100±5900, P = 0.179, N = 46; 20°C/18 h: −240±4600, P = 0.958, N = 46; 27°C/12 h: 5800±5400, P = 0.288, N = 47; 27°C/18 h: −13800±7900, P = 0.086, N = 46;). Only 1 out of 8 within-group correlations was significant, showing a positive correlation between growth rate and chill-coma recovery time (Table S12). Table S4: Results of a linear model for the effects of rearing temperature (RT), photoperiod (PhP), and sex on chill-coma recovery (CCR) in Protophormia terranovae. Growth rate (GR) was included as continuous variable. Significant p-values are given in bold. (DOCX) [file pone.0062434.s004.docx]

**Table S4**

|  |  |  |  |  |  |
| --- | --- | --- | --- | --- | --- |
| **Experiment 11** | **Source** | **MS** | **DF** | **F** | **P** |
| CCR | RT | 6631917 | 1 | 13.73 | **< 0.001** |
|  | PhP | 2780630 | 1 | 5.75 | **0.017** |
|  | Sex | 2073520 | 1 | 4.29 | **0.040** |
|  | RT*PhP | 5093 | 1 | 0.01 | 0.918 |
|  | RT*Sex | 3696 | 1 | < 0.00 | 0.930 |
|  | PhP*Sex | 1021707 | 1 | 2.11 | 0.147 |
|  | RT*PhP*Sex | 97726 | 1 | 0.20 | 0.653 |
|  | GR | 23982 | 1 | 0.04 | 0.824 |
|  | Error | 482768 | 179 |  |  |
