# Supplementary material for: No Trade-Off between Growth Rate and Temperature Stress Resistance in Four Insect Species
Source: PLoS One. 2013 Apr 30;8(4):e62434. doi: 10.1371/journal.pone.0062434 (PMC3640073; doi:10.1371/journal.pone.0062434)
Supplement: Table S5 — Experiments 1 and 2 (Bicyclus anynana). Results of linear (mixed) models including interactions with the continuous variable growth rate (GR) for the butterfly Bicyclus anynana used in experiments 1 and 2. In experiment 1, effects of inbreeding level (Inbreeding), sex and block (random factor) on chill-coma recovery (CCR) and heat knock-down time (HKD) were investigated. In experiment 2 two separate analyses were used (see Methods). First, the effects of selection regime (Selection), replicate line (nested within selection regime, random), inbreeding level (Inbreeding), rearing temperature (RT), acclimation temperature (AT), and sex (experiment 2A), and second the effects of selection regime (Selection), replicate line (nested within selection regime, random), inbreeding level (Inbreeding), acclimation temperature (AT), adult feeding treatment (Food), and sex on chill-coma recovery time were investigated (experiment 2B). Growth rate (GR) was included as continuous variable throughout. Significant p-values are given in bold. (DOCX) [file pone.0062434.s005.docx]

**Table S5**

|  |  |  |  |  |  |  |
| --- | --- | --- | --- | --- | --- | --- |
| **Experiment 1** | **Source** | **MS** | **DF** | **F** | **P** |  |
| CCR | Inbreeding | 1809358 | 2 | 2.15 | 0.118 |  |
|  | Sex | 276398 | 1 | 0.33 | 0.567 |  |
|  | Block | 1463116 | 28 | 1.74 | **0.013** |  |
|  | Inbreeding*Sex | 425750 | 2 | 0.51 | 0.604 |  |
|  | Inbreeding*GR | 1471439 | 2 | 1.75 | 0.176 |  |
|  | Sex*GR | 103922 | 1 | 0.12 | 0.726 |  |
|  | Inbreeding*Sex*GR | 336315 | 2 | 0.40 | 0.671 |  |
|  | GR | 9502933 | 1 | 11.28 | **0.001** |  |
|  | Error | 842442 | 344 |  |  |  |
| HKD | Inbreeding | 1644351 | 2 | 2.95 | 0.054 |  |
|  | Sex | 3164850 | 1 | 5.67 | **0.018** |  |
|  | Inbreeding*Sex | 68828 | 2 | 0.12 | 0.884 |  |
|  | Inbreeding*GR | 1565136 | 2 | 2.80 | 0.062 |  |
|  | Sex*GR | 3948371 | 1 | 7.08 | **0.008** |  |
|  | Inbreeding*Sex*GR | 85665 | 1 | 0.15 | 0.858 |  |
|  | GR | 6755836 | 1 | 12.11 | **0.001** |  |
|  | Error | 558101 | 276 |  |  |  |
| **Experiment 2A** | Source | **MS** | **DF** | **F** | **P** |  |
| CCR | Selection | 338.0 | 1 | 2.43 | 0.119 |  |
|  | Repl.[(Sel.] | 981.7 | 2 | 7.26 | **0.001** |  |
|  | Inbreeding | 162.7 | 2 | 1.20 | 0.300 |  |
|  | AT | 2544.7 | 1 | 18.82 | **< 0.001** |  |
|  | RT | 811.2 | 1 | 6.00 | **0.014** |  |
|  | Sex | 92.5 | 1 | 0.68 | 0.408 |  |
|  | Sel.*Inbreed. | 78.4 | 2 | 0.58 | 0.560 |  |
|  | Sel.*AT | 417.9 | 1 | 3.09 | 0.079 |  |
|  | Inbreed.*AT | 392.3 | 2 | 2.90 | 0.055 |  |
|  | Sel.*RT | 18.8 | 1 | 0.13 | 0.708 |  |
|  | Inbreed.*RT | 1003.1 | 2 | 7.42 | **0.001** |  |
|  | AT*RT | 1072.0 | 1 | 7.93 | **0.005** |  |
|  | Sel.*Sex | 167.7 | 1 | 1.24 | 0.265 |  |
|  | Inbreed.*Sex | 83.6 | 2 | 0.61 | 0.539 |  |
|  | AT*Sex | 675.6 | 1 | 4.99 | **0.025** |  |
|  | RT*Sex | 133.9 | 1 | 0.99 | 0.320 |  |
|  | Sel.*GR | 14.4 | 1 | 0.10 | 0.743 |  |
|  | Inbreed.*GR | 46.3 | 2 | 0.34 | 0.710 |  |
|  | AT*GR | 672.3 | 1 | 4.97 | **0.026** |  |
|  | RT*GR | 334.5 | 1 | 2.47 | 0.116 |  |
|  | Sex*GR | 1.8 | 1 | 0.01 | 0.908 |  |
|  | Sel.*Inbreed.*AT | 295.1 | 2 | 2.18 | 0.113 |  |
|  | Sel.*Inbreed.*RT | 106.2 | 2 | 0.78 | 0.456 |  |
|  | Sel.*AT*RT | 113.1 | 1 | 0.83 | 0.360 |  |
|  | Inbreed.*AT*RT | 1217.8 | 2 | 9.01 | **< 0.001** |  |
|  | Sel.*Inbreed.*Sex | 341.8 | 2 | 2.52 | 0.080 |  |
|  | Sel.*AT*Sex | 88.3 | 1 | 0.65 | 0.419 |  |
|  | Inbreed.*AT*Sex | 53.9 | 2 | 0.39 | 0.671 |  |
|  | Sel.*RT*Sex | 136.2 | 1 | 1.00 | 0.315 |  |
|  | Inbreed.*RT*Sex | 10.1 | 2 | 0.07 | 0.928 |  |
|  | AT*RT*Sex | 265.2 | 1 | 1.96 | 0.161 |  |
|  | Sel.*Inbreed.*GR | 191.8 | 2 | 1.41 | 0.242 |  |
|  | Sel.*AT*GR | 240.4 | 1 | 1.77 | 0.182 |  |
|  | Inbreed.*AT*GR | 217.9 | 2 | 1.61 | 0.200 |  |
|  | Sel.*RT*GR | 7.9 | 1 | 0.05 | 0.808 |  |
|  | Inbreed.*RT*GR | 464.7 | 2 | 3.43 | **0.032** |  |
|  | AT*RT*GR | 1124.5 | 1 | 8.32 | **0.004** |  |
|  | Sel.*Sex*GR | 233.0 | 1 | 1.72 | 0.189 |  |
|  | Inbreed.*Sex*GR | 117.1 | 2 | 0.86 | 0.421 |  |
|  | AT*Sex*GR | 464.4 | 1 | 3.43 | 0.064 |  |
|  | RT*Sex*GR | 18.0 | 1 | 0.13 | 0.715 |  |
|  | Sel.*Inbreed.*AT*RT | 136.9 | 2 | 1.01 | 0.363 |  |
|  | Sel.*Inbreed.*AT*Sex | 386.5 | 2 | 2.85 | 0.057 |  |
|  | Sel.*Inbreed.*RT*Sex | 15.8 | 2 | 0.11 | 0.890 |  |
|  | Sel.*AT*RT*Sex | 251.6 | 1 | 1.86 | 0.172 |  |
|  | Inbreed.*AT*RT*Sex | 239.0 | 2 | 1.76 | 0.171 |  |
|  | Sel.*Inbreed.*AT*GR | 420.5 | 2 | 3.11 | **0.045** |  |
|  | Sel.*Inbreed.*RT*GR | 43.9 | 2 | 0.32 | 0.723 |  |
|  | Sel.*AT*RT*GR | 44.6 | 1 | 0.33 | 0.566 |  |
|  | Inbreed.*AT*RT*GR | 647.2 | 2 | 4.78 | **0.008** |  |
|  | Sel.*Inbreed.*Sex*GR | 174.5 | 2 | 1.29 | 0.275 |  |
|  | Sel.*AT*Sex*GR | 143.7 | 1 | 1.06 | 0.302 |  |
|  | Inbreed.*AT*Sex*GR | 67.1 | 2 | 0.49 | 0.608 |  |
|  | Sel.*RT*Sex*GR | 53.5 | 1 | 0.39 | 0.529 |  |
|  | Inbreed.*RT*Sex*GR | 9.5 | 2 | 0.07 | 0.932 |  |
|  | AT*RT*Sex*GR | 95.8 | 1 | 0.70 | 0.400 |  |
|  | Sel.*Inbreed.*AT*RT*Sex | 5.5 | 2 | 0.04 | 0.960 |  |
|  | Sel.*Inbreed.*AT*RT*GR | 78.3 | 2 | 0.57 | 0.560 |  |
|  | Sel.*Inbreed.*AT*Sex*GR | 213.6 | 2 | 1.58 | 0.206 |  |
|  | Sel.*Inbreed.*RT*Sex*GR | 24.7 | 2 | 0.18 | 0.833 |  |
|  | Sel.*AT*RT*Sex*GR | 108.8 | 1 | 0.80 | 0.370 |  |
|  | Inbreed.*AT*RT*Sex*GR | 235.3 | 2 | 1.74 | 0.175 |  |
|  | Sel.*Inbreed.*AT*RT*Sex*GR | 13.8 | 2 | 0.10 | 0.903 |  |
|  | GR | 0.7 | 1 | <0.00 | 0.941 |  |
|  | Error | 135.1 | 3144 |  |  |  |
| **Experiment 2B** | Source | **MS** | **DF** | **F** | **P** |  |
| CCR | Selection | 275.3 | 1 | 1.35 | 0.246 |  |
|  | Repl.[Sel.] | 2537.3 | 2 | 13.43 | **< 0.001** |  |
|  | Inbreeding | 292.8 | 2 | 1.55 | 0.212 |  |
|  | AT | 8.6 | 1 | 0.04 | 0.831 |  |
|  | Food | 578.7 | 1 | 3.06 | 0.080 |  |
|  | Sex | 937.1 | 1 | 4.96 | **0.026** |  |
|  | Sel.*Inbreed. | 12.6 | 2 | 0.06 | 0.935 |  |
|  | Sel.*AT | 1094.5 | 1 | 5.79 | **0.016** |  |
|  | Inbreed.*AT | 1827.9 | 2 | 9.68 | **< 0.001** |  |
|  | Sel.*Food | 626.5 | 1 | 3.31 | 0.069 |  |
|  | Inbreed.*Food | 423.6 | 2 | 2.24 | 0.106 |  |
|  | AT*Food | 30.2 | 1 | 0.16 | 0.689 |  |
|  | Sel.*Sex | 11.8 | 1 | 0.06 | 0.802 |  |
|  | Inbreed.*Sex | 343.3 | 2 | 1.81 | 0.162 |  |
|  | AT*Sex | 33.5 | 1 | 0.17 | 0.673 |  |
|  | Food*Sex | 5377.0 | 1 | 28.47 | **< 0.001** |  |
|  | Sel.*GR | 26.4 | 1 | 0.14 | 0.708 |  |
|  | Inbreed.*GR | 168.5 | 2 | 0.89 | 0.410 |  |
|  | AT*GR | 571.8 | 1 | 3.02 | 0.082 |  |
|  | Food*GR | 622.4 | 1 | 3.29 | 0.070 |  |
|  | Sex*GR | 994.9 | 1 | 5.26 | **0.022** |  |
|  | Sel.*Inbreed.*AT | 790.7 | 2 | 4.18 | **0.015** |  |
|  | Sel.*Inbreed.*Food | 76.8 | 2 | 0.40 | 0.666 |  |
|  | Sel.*AT*Food | 163.3 | 1 | 0.86 | 0.352 |  |
|  | Inbreed.*AT*Food | 904.8 | 2 | 4.79 | **0.008** |  |
|  | Sel.*Inbreed.*Sex | 341.8 | 2 | 1.81 | 0.164 |  |
|  | Sel.*AT*Sex | 898.1 | 1 | 4.75 | **0.029** |  |
|  | Inbreed.*AT*Sex | 297.6 | 2 | 1.57 | 0.207 |  |
|  | Sel.*Food*Sex | 12.1 | 1 | 0.06 | 0.800 |  |
|  | Inbreed.*Food*Sex | 130.5 | 2 | 0.69 | 0.501 |  |
|  | AT*Food*Sex | 643.5 | 1 | 3.40 | 0.065 |  |
|  | Sel.*Inbreed.*GR | 29.4 | 2 | 0.15 | 0.856 |  |
|  | Sel.*AT*GR | 1194.6 | 1 | 6.32 | **0.012** |  |
|  | Inbreed.*AT*GR | 1768.2 | 2 | 9.36 | **< 0.001** |  |
|  | Sel.*Food*GR | 632.1 | 1 | 3.34 | 0.067 |  |
|  | Inbreed.*Food*GR | 464.7 | 2 | 2.46 | 0.085 |  |
|  | AT*Food*GR | 30.7 | 1 | 0.16 | 0.687 |  |
|  | Sel.*Sex*GR | 10.7 | 1 | 0.05 | 0.812 |  |
|  | Inbreed.*Sex*GR | 383.8 | 2 | 2.03 | 0.131 |  |
|  | AT*Sex*GR | < 0.0 | 1 | < 0.00 | 0.983 |  |
|  | Food*Sex*GR | 5290.8 | 1 | 28.02 | **< 0.001** |  |
|  | Sel.*Inbreed.*AT*Food | 13.8 | 2 | 0.07 | 0.929 |  |
|  | Sel.*Inbreed.*AT*Sex | 13.0 | 2 | 0.06 | 0.933 |  |
|  | Sel.*Inbreed.*Food*Sex | 587.9 | 2 | 3.11 | **0.045** |  |
|  | Sel.*AT*Food*Sex | 0.3 | 1 | < 0.00 | 0.967 |  |
|  | Inbreed.*AT*Food*Sex | 84.5 | 2 | 0.44 | 0.639 |  |
|  | Sel.*Inbreed.*AT*GR | 846.8 | 2 | 4.48 | **0.011** |  |
|  | Sel.*Inbreed.*Food*GR | 67.5 | 2 | 0.35 | 0.699 |  |
|  | Sel.*AT*Food*GR | 228.7 | 1 | 1.21 | 0.271 |  |
|  | Inbreed.*AT*Food*GR | 826.7 | 2 | 4.37 | **0.013** |  |
|  | Sel.*Inbreed.*Sex*GR | 336.3 | 2 | 1.78 | 0.169 |  |
|  | Sel.*AT*Sex*GR | 877.6 | 1 | 4.64 | **0.031** |  |
|  | Inbreed.*AT*Sex*GR | 313.7 | 2 | 1.66 | 0.190 |  |
|  | Sel.*Food*Sex*GR | 25.1 | 1 | 0.13 | 0.715 |  |
|  | Inbreed.*Food*Sex*GR | 133.0 | 2 | 0.70 | 0.494 |  |
|  | AT*Food*Sex*GR | 530.0 | 1 | 2.80 | 0.094 |  |
|  | Sel.*Inbreed.*AT*Food*Sex | 51.2 | 2 | 0.27 | 0.762 |  |
|  | Sel.*Inbreed.*AT*Food*GR | 9.4 | 2 | 0.04 | 0.951 |  |
|  | Sel.*Inbreed.*AT*Sex*GR | 9.8 | 2 | 0.05 | 0.949 |  |
|  | Sel.*Inbreed.*Food*Sex*GR | 624.0 | 2 | 3.30 | **0.037** |  |
|  | Sel.*AT*Food*Sex*GR | < 0.0 | 1 | < 0.00 | **< 0.001** |  |
|  | Inbreed.*AT*Food*Sex*GR | 87.1 | 2 | 0.46 | 0.630 |  |
|  | Sel.*Inbreed.*AT*Food*Sex*GR | 44.5 | 2 | 0.23 | 0.790 |  |
|  | GR | 653.2 | 1 | 3.45 | 0.063 |  |
|  | Error | 188.8 | 2993 |  |  |  |
