# Supplementary material for: No Trade-Off between Growth Rate and Temperature Stress Resistance in Four Insect Species
Source: PLoS One. 2013 Apr 30;8(4):e62434. doi: 10.1371/journal.pone.0062434 (PMC3640073; doi:10.1371/journal.pone.0062434)
Supplement: Table S6 — Experiments 3–7 (Lycaena tityrus). Results of linear (mixed) models including interactions with the continuous variable growth rate (GR) for the butterfly Lycaena tityrus used in experiments 3–7. In experiment 3 the effects of rearing temperature (RT), acclimation temperature (AT), family (random factor), and sex on chill-coma recovery (CCR) were investigated; in experiment 4 the effects of mean temperature (Temperature), temperature variation (Variation), and sex on chill-coma recovery and heat knock-down time (HKD), respectively, were investigated; in experiment 5 the effects of altitude, replicate population (nested within altitude, random), rearing temperature (RT), and sex on chill-coma recovery and heat knock-down time, respectively, were investigated; in experiment 6 the effects of altitude, replicate population (nested within altitude, random), and sex on chill-coma recovery and heat knock-down time, respectively, were investigated; in experiment 7 the effect of PGI genotype, rearing temperature (RT), and sex on chill-coma recovery and heat knock-down time, respectively, were investigated. Growth rate (GR) was included as continuous variable throughout. Significant p-values are given in bold. (DOCX) [file pone.0062434.s006.docx]

**Table S6**

|  |  |  |  |  |  |
| --- | --- | --- | --- | --- | --- |
| **Experiment 3** | **Source** | **MS** | **DF** | **F** | **P** |
| CCR | RT | 292701 | 1 | 0.75 | 0.385 |
|  | AT | 1251767 | 1 | 3.23 | 0.073 |
|  | Family | 871413 | 4 | 2.24 | 0.064 |
|  | Sex | 1362511 | 1 | 3.51 | 0.062 |
|  | RT*AT | 1280437 | 1 | 3.30 | 0.070 |
|  | RT*Sex | 1700398 | 1 | 4.38 | **0.037** |
|  | AT*Sex | 1059570 | 1 | 2.73 | 0.099 |
|  | RT*GR | 428663 | 1 | 1.10 | 0.294 |
|  | AT*GR | 655556 | 1 | 1.69 | 0.194 |
|  | Sex*GR | 806176 | 1 | 2.08 | 0.150 |
|  | RT*AT*Sex | 1560515 | 1 | 4.02 | **0.046** |
|  | RT*AT*GR | 991525 | 1 | 2.55 | 0.111 |
|  | RT*Sex*GR | 960671 | 1 | 2.47 | 0.116 |
|  | AT*Sex*GR | 439058 | 1 | 1.13 | 0.288 |
|  | RT*AT*Sex*GR | 1152367 | 1 | 2.97 | 0.086 |
|  | GR | 219166 | 1 | 0.56 | 0.453 |
|  | Error | 387501 | 322 | 2.97 | 0.086 |
| **Experiment 4** | **Source** | **MS** | **DF** | **F** | **P** |
| CCR | Temperature | 49964.1 | 1 | 0.65 | 0.419 |
|  | Variation | 68638.8 | 1 | 0.90 | 0.343 |
|  | Sex | 298430.2 | 1 | 3.91 | **0.049** |
|  | Temp*Var. | 292047.9 | 1 | 3.82 | 0.051 |
|  | Temp.*Sex | 118947.5 | 1 | 1.55 | 0.213 |
|  | Var.*Sex | 175573.0 | 1 | 2.30 | 0.130 |
|  | Temp.*GR | 135827.3 | 1 | 1.78 | 0.183 |
|  | Var.*GR | 95225.6 | 1 | 1.24 | 0.265 |
|  | Sex*GR | 297099.8 | 1 | 3.89 | **0.049** |
|  | Temp.*Var.*Sex | 162314.4 | 1 | 2.12 | 0.145 |
|  | Temp.*Var.*GR | 210592.0 | 1 | 2.76 | 0.097 |
|  | Temp.*Sex*GR | 146820.1 | 1 | 1.92 | 0.166 |
|  | Var.*Sex*GR | 186214.1 | 1 | 2.44 | 0.119 |
|  | Temp.*Var.*Sex*GR | 156613.3 | 1 | 2.05 | 0.153 |
|  | GR | 857749.5 | 1 | 11.23 | **0.001** |
|  | Error | 76259.8 | 361 |  |  |
| HKD | Temperature | 8722320 | 1 | 7.73 | **0.006** |
|  | Variation | 2352881 | 1 | 2.09 | 0.149 |
|  | Sex | 5099485 | 1 | 4.53 | **0.034** |
|  | Temp.*Var. | 2108458 | 1 | 1.87 | 0.172 |
|  | Temp.*Sex | 60809 | 1 | 0.05 | 0.816 |
|  | Var.*Sex | 781553 | 1 | 0.69 | 0.405 |
|  | Temp.*GR | 2902194 | 1 | 2.58 | 0.109 |
|  | Var.*GR | 615451 | 1 | 0.54 | 0.460 |
|  | Sex*GR | 3859846 | 1 | 3.43 | 0.065 |
|  | Temp.*Var.*Sex | 2415766 | 1 | 2.15 | 0.143 |
|  | Temp.*Var.*GR | 769690 | 1 | 0.68 | 0.408 |
|  | Temp.*Sex*GR | 233965 | 1 | 0.20 | 0.648 |
|  | Var.*Sex*GR | 68126 | 1 | 0.06 | 0.806 |
|  | Temp.*Var.*Sex*GR | 1482325 | 1 | 1.31 | 0.251 |
|  | GR | 1878616 | 1 | 1.67 | 0.197 |
|  | Error | 1123369 | 354 |  |  |
| **Experiment 5** | **Source** | **MS** | **DF** | **F** | **P** |
| CCR | Altitude | 273.5 | 1,3 | < 0.00 | 0.938 |
|  | Repl.[Alt.] | 7927.9 | 2,387 | 0.17 | 0.840 |
|  | Sex | 41368.6 | 1,387 | 0.90 | 0.341 |
|  | RT | 5522.2 | 1,387 | 0.12 | 0.728 |
|  | Alt.*Sex | 24235.8 | 1,387 | 0.53 | 0.466 |
|  | Alt.*RT | 157369.0 | 1,387 | 3.45 | 0.064 |
|  | Sex*RT | 18521.6 | 1,387 | 0.40 | 0.524 |
|  | Alt.*GR | 188.8 | 1,387 | < 0.00 | 0.949 |
|  | Sex*GR | 58029.2 | 1,387 | 1.27 | 0.260 |
|  | RT*GR | 8381.5 | 1,387 | 0.18 | 0.668 |
|  | Alt.*Sex*RT | 17624.4 | 1,387 | 0.38 | 0.534 |
|  | Alt.*Sex*GR | 20683.9 | 1,387 | 0.45 | 0.501 |
|  | Alt.*RT*GR | 149061.8 | 1,387 | 3.27 | 0.071 |
|  | Sex*RT*GR | 31660.2 | 1,387 | 0.69 | 0.405 |
|  | Alt.*Sex*RT*GR | 5274.5 | 1,387 | 0.11 | 0.734 |
|  | GR | 46168.2 | 1,387 | 1.01 | 0.315 |
|  | Error | 45564.7 | 387 |  |  |
| HKD | Altitude | 111144.4 | 1,3 | 2.33 | 0.127 |
|  | Repl.[Alt] | 75224.9 | 2,383 | 1.59 | 0.205 |
|  | Sex | 40556.7 | 1,383 | 0.85 | 0.355 |
|  | RT | 150516.0 | 1,383 | 3.18 | 0.075 |
|  | Alt.*Sex | 15395.3 | 1,383 | 0.32 | 0.568 |
|  | Alt.*RT | 14669.3 | 1,383 | 0.31 | 0.578 |
|  | Sex*RT | 60992.3 | 1,383 | 1.29 | 0.257 |
|  | Alt.*GR | 140354.2 | 1,383 | 2.96 | 0.086 |
|  | Sex*GR | 10664.8 | 1,383 | 0.22 | 0.635 |
|  | RT*GR | 182136.3 | 1,383 | 3.85 | 0.050 |
|  | Alt.*Sex*RT | 1242.1 | 1,383 | 0.02 | 0.871 |
|  | Alt.*Sex*GR | 20465.4 | 1,383 | 0.43 | 0.511 |
|  | Alt.*RT*GR | 9314.8 | 1,383 | 0.19 | 0.657 |
|  | Sex*RT*GR | 40693.1 | 1,383 | 0.86 | 0.354 |
|  | Alt.*Sex*RT*GR | 25.1 | 1,383 | < 0.00 | 0.982 |
|  | GR | 50268.4 | 1,383 | 1.06 | 0.303 |
|  | Error | 47258.0 | 383 |  |  |
| **Experiment 6** | Source | **MS** | **DF** | **F** | **P** |
| CCR | Altitude | 247132.3 | 2,3 | 9.67 | **< 0.001** |
|  | Repl.[Alt.] | 117909.7 | 3,266 | 4.70 | **0.003** |
|  | Sex | 22044.6 | 1,266 | 0.87 | 0.349 |
|  | Alt.*Sex | 67501.8 | 2,266 | 2.69 | 0.070 |
|  | Alt.*GR | 169399.3 | 2,266 | 6.75 | **0.001** |
|  | Sex*GR | 19752.3 | 1,266 | 0.78 | 0.376 |
|  | Alt.*Sex*GR | 65574.7 | 2,266 | 2.61 | 0.075 |
|  | GR | 16505.3 | 1,266 | 0.65 | 0.418 |
|  | Error | 25067.9 | 266 |  |  |
| HKD | Altitude | 432900.3 | 2,3 | 5.98 | **0.003** |
|  | Repl.[Alt.] | 47159.7 | 3,266 | 0.65 | 0.583 |
|  | Sex | 83979.3 | 1,266 | 1.15 | 0.283 |
|  | Alt.*Sex | 463289.1 | 2,266 | 6.39 | **0.002** |
|  | Alt.*GR | 358209.0 | 2,266 | 4.94 | **0.008** |
|  | Sex*GR | 33710.6 | 1,266 | 0.46 | 0.496 |
|  | Alt.*Sex*GR | 477043.6 | 2,266 | 6.58 | **0.002** |
|  | GR | 338.9 | 1,266 | < 0.00 | 0.946 |
|  | Error | 72477.9 | 266 |  |  |
| **Experiment 7** | **Source** | **MS** | **DF** | **F** | **P** |
| CCR | Genotype | 80950.0 | 3 | 1.08 | 0.354 |
|  | Temperature | 10968.8 | 1 | 0.14 | 0.701 |
|  | Sex | 105512.1 | 1 | 1.41 | 0.234 |
|  | GT*Temp. | 4034.8 | 3 | 0.05 | 0.983 |
|  | GT*Sex | 61257.3 | 3 | 0.82 | 0.482 |
|  | Temp.*Sex | 12987.1 | 1 | 0.17 | 0.676 |
|  | GT*GR | 78923.7 | 3 | 1.05 | 0.366 |
|  | Temp.*GR | 19976.4 | 1 | 0.26 | 0.605 |
|  | Sex*GR | 117624.2 | 1 | 1.57 | 0.209 |
|  | GT*Temp.*Sex | 17093.6 | 3 | 0.22 | 0.876 |
|  | GT*Temp.*GR | 5931.6 | 3 | 0.07 | 0.971 |
|  | GT*Sex*GR | 57691.5 | 3 | 0.77 | 0.509 |
|  | Temp.*Sex*GR | 28401.7 | 1 | 0.38 | 0.537 |
|  | GT*Temp.*Sex*GR | 14937.6 | 3 | 0.20 | 0.896 |
|  | GR | 12914.5 | 1 | 0.17 | 0.677 |
|  | Error | 74492.5 | 618 |  |  |
| HKD | Genotype | 70652.9 | 3 | 1.66 | 0.173 |
|  | Temperature | 11855.6 | 1 | 0.27 | 0.597 |
|  | Sex | 10772.3 | 1 | 0.25 | 0.614 |
|  | GT*Temp. | 26687.1 | 3 | 0.62 | 0.596 |
|  | GT*Sex | 156557.9 | 3 | 3.69 | **0.012** |
|  | Temp.*Sex | 815.7 | 1 | 0.01 | 0.890 |
|  | GT*GR | 65489.2 | 3 | 1.54 | 0.202 |
|  | Temp.*GR | 1482.7 | 1 | 0.03 | 0.852 |
|  | Sex*GR | 45913.0 | 1 | 1.08 | 0.298 |
|  | GT*Temp.*Sex | 62952.5 | 3 | 1.48 | 0.217 |
|  | GT*Temp.*GR | 19250.8 | 3 | 0.45 | 0.714 |
|  | GT*Sex*GR | 143284.8 | 3 | 3.38 | **0.018** |
|  | Temp.*Sex*GR | 1039.5 | 1 | 0.02 | 0.876 |
|  | GT*Temp.*Sex*GR | 49807.2 | 3 | 1.17 | 0.318 |
|  | GR | 453791.6 | 1 | 10.70 | **0.001** |
|  | Error | 42389.5 | 622 |  |  |
