# Supplementary material for: No Trade-Off between Growth Rate and Temperature Stress Resistance in Four Insect Species
Source: PLoS One. 2013 Apr 30;8(4):e62434. doi: 10.1371/journal.pone.0062434 (PMC3640073; doi:10.1371/journal.pone.0062434)
Supplement: Table S7 — Experiments 8–10 (Pieris napi). Results of linear models including interactions with the continuous variable growth rate (GR) for the butterfly Pieris napi used in experiments 8–10. In experiment 8 the effects of thermal regime (TR) and sex on chill-coma recovery (CCR) and heat knock-down time (HKD), respectively, were investigated; in experiment 9 the effects of rearing temperature (RT), block and sex on chill-coma recovery and heat knock-down time and in experiment 10 the effects of rearing temperature (RT) and sex on chill-coma recovery and heat knock-down time, respectively, were investigated. Growth rate (GR) was included as continuous variable throughout. Significant p-values are given in bold. (DOCX) [file pone.0062434.s007.docx]

**Table S7**

|  | |  | |  | |  | |  | |  | |
| --- | --- | --- | --- | --- | --- | --- | --- | --- | --- | --- | --- |
| **Experiment 8** | **Source** | | **MS** | | **DF** | | **F** | | **P** | |  |
| CCR | TR | | 118561.3 | | 3 | | 5.01 | | **0.002** | |  |
|  | Sex | | 9693.4 | | 1 | | 0.41 | | 0.523 | |  |
|  | TR*Sex | | 12604.5 | | 3 | | 0.53 | | 0.660 | |  |
|  | TR*GR | | 73718.7 | | 3 | | 3.11 | | **0.027** | |  |
|  | Sex*GR | | 11609.9 | | 1 | | 0.49 | | 0.484 | |  |
|  | TR*Sex*GR | | 15302.0 | | 3 | | 0.64 | | 0.585 | |  |
|  | GR | | 9166.5 | | 1 | | 0.38 | | 0.534 | |  |
|  | Error | | 23639.6 | | 240 | |  | |  | |  |
| HKD | TR | | 570233 | | 3 | | 2.24 | | 0.084 | |  |
|  | Sex | | 5086 | | 1 | | 0.02 | | 0.888 | |  |
|  | TR*Sex | | 220167 | | 3 | | 0.86 | | 0.459 | |  |
|  | TR*GR | | 585716 | | 3 | | 2.30 | | 0.078 | |  |
|  | Sex*GR | | 2383 | | 1 | | < 0.00 | | 0.923 | |  |
|  | TR*Sex*GR | | 229188 | | 3 | | 0.90 | | 0.441 | |  |
|  | GR | | 328236 | | 1 | | 1.29 | | 0.257 | |  |
|  | Error | | 253913 | | 215 | |  | |  | |  |
| **Experiment 9** | **Source** | | **MS** | | **DF** | | **F** | | **P** | |  |
| CCR | RT | | 66605.4 | | 1 | | 0.45 | | 0.499 | |  |
|  | Sex | | 2082.5 | | 1 | | 0.01 | | 0.905 | |  |
|  | Block | | 381580.0 | | 26 | | 2.62 | | **< 0.001** | |  |
|  | RT*Sex | | 305021.5 | | 1 | | 2.10 | | 0.149 | |  |
|  | RT*GR | | 64814.9 | | 1 | | 0.44 | | 0.505 | |  |
|  | Sex*GR | | 1016.0 | | 1 | | 0.01 | | 0.933 | |  |
|  | RT*Sex*GR | | 343244.9 | | 1 | | 2.36 | | 0.126 | |  |
|  | GR | | 7746.2 | | 1 | | 0.05 | | 0.818 | |  |
|  | Error | | 145122.5 | | 165 | |  | |  | |  |
| HKD | RT | | 42350 | | 1 | | 0.73 | | 0.394 | |  |
|  | Sex | | 9 | | 1 | | < 0.00 | | 0.990 | |  |
|  | Block | | 129808 | | 22 | | 2.24 | | **0.003** | |  |
|  | RT*Sex | | 32945 | | 1 | | 0.56 | | 0.452 | |  |
|  | RT*GR | | 289 | | 1 | | < 0.00 | | 0.944 | |  |
|  | Sex*GR | | 3292 | | 1 | | 0.05 | | 0.812 | |  |
|  | RT*Sex*GR | | 38066 | | 1 | | 0.65 | | 0.419 | |  |
|  | GR | | 46392 | | 1 | | 0.80 | | 0.372 | |  |
|  | Error | | 57907 | | 134 | |  | |  | |  |
| **Experiment 10** | **Source** | | **MS** | | **DF** | | **F** | | **P** | |  |
| CCR | RT | | 162793 | | 1 | | 1.31 | | 0.252 | |  |
|  | Sex | | 144039 | | 1 | | 1.16 | | 0.282 | |  |
|  | RT*Sex | | 108489 | | 1 | | 0.87 | | 0.350 | |  |
|  | RT*GR | | 78916 | | 1 | | 0.63 | | 0.425 | |  |
|  | Sex*GR | | 57540 | | 1 | | 0.46 | | 0.496 | |  |
|  | RT*Sex*GR | | 62734 | | 1 | | 0.50 | | 0.477 | |  |
|  | GR | | 21093 | | 1 | | 0.17 | | 0.680 | |  |
|  | Error | | 123894 | | 353 | |  | |  | |  |
| HKD | RT | | 53438 | | 1 | | 0.77 | | 0.380 | |  |
|  | Sex | | 153631 | | 1 | | 2.22 | | 0.137 | |  |
|  | RT*Sex | | 12041 | | 1 | | 0.17 | | 0.677 | |  |
|  | RT*GR | | 172 | | 1 | | < 0.00 | | 0.960 | |  |
|  | Sex*GR | | 128272 | | 1 | | 1.85 | | 0.174 | |  |
|  | RT*Sex*GR | | 34209 | | 1 | | 0.49 | | 0.482 | |  |
|  | GR | | 59680 | | 1 | | 0.86 | | 0.353 | |  |
|  | Error | | 69051 | | 343 | |  | |  | |  |
