# Supplementary material for: No Trade-Off between Growth Rate and Temperature Stress Resistance in Four Insect Species
Source: PLoS One. 2013 Apr 30;8(4):e62434. doi: 10.1371/journal.pone.0062434 (PMC3640073; doi:10.1371/journal.pone.0062434)
Supplement: Table S8 — Experiment 11 (Protophormia terraenovae). Results of an linear model including interactions with the continuous variable growth rate (GR) for the fly Protophormia terranovae used in experiment 11. The effects of rearing temperature (RT), photoperiod (PhP), sex, and the continuous variable growth rate on chill-coma recovery (CCR) were investigated. Significant p-values are given in bold. (DOCX) [file pone.0062434.s008.docx]

**Table S8**

|  |  |  |  |  |  |
| --- | --- | --- | --- | --- | --- |
| **Experiment 11** | **Source** | **MS** | **DF** | **F** | **P** |
| CCR | RT | 261234 | 1 | 0.56 | 0.452 |
|  | PhP | 1890055 | 1 | 4.11 | **0.044** |
|  | Sex | 202234 | 1 | 0.44 | 0.508 |
|  | RT*PhP | 1795268 | 1 | 3.91 | 0.050 |
|  | RT*Sex | 975998 | 1 | 2.12 | 0.147 |
|  | PhP*Sex | 144623 | 1 | 0.31 | 0.575 |
|  | RT*GR | 354515 | 1 | 0.77 | 0.381 |
|  | PhP*GR | 2025948 | 1 | 4.41 | **0.037** |
|  | Sex*GR | 135650 | 1 | 0.29 | 0.587 |
|  | RT*PhP*Sex | 176743 | 1 | 0.38 | 0.536 |
|  | RT*PhP*GR | 1858923 | 1 | 4.05 | **0.046** |
|  | RT*Sex*GR | 886753 | 1 | 1.93 | 0.166 |
|  | PhP*Sex*GR | 156278 | 1 | 0.34 | 0.560 |
|  | RT*PhP*Sex*GR | 186542 | 1 | 0.40 | 0.525 |
|  | GR | 203019 | 1 | 0.44 | 0.507 |
|  | Error | 458831 | 172 |  |  |
