# Supplementary material for: No Trade-Off between Growth Rate and Temperature Stress Resistance in Four Insect Species
Source: PLoS One. 2013 Apr 30;8(4):e62434. doi: 10.1371/journal.pone.0062434 (PMC3640073; doi:10.1371/journal.pone.0062434)
Supplement: Table S9 — Experiments 1 and 2 (Bicyclus anynana). Within-group correlations between growth rate and temperature stress resistance (chill coma recovery, CCR and/or heat knock down time, HKD) for the butterfly Bicyclus anynana in experiment 1 (N = 6 correlations per trait) and experiment 2 (N = 70 correlations). Inbreeding (Inb) 1 = inbreeding level 1, Inbreeding (Inb) 2 = inbreeding level 2, Inbreeding (Inb) 0 = outbred controls; Sel 0 = unselected control lines, Sel 1 = cold selected lines; RT = rearing temperature (20 or 27°C); AT = acclimation temperature (20 or 27°C); F 0 = starvation, F 1 = no starvation; M = male, F = female. Significant correlations are given in bold. (DOCX) [file pone.0062434.s009.docx]

**Table S9**

| **Exp.** | **Trait** | **Treatment group** | **Sex** | **R** | **P** | **N** |
| --- | --- | --- | --- | --- | --- | --- |
| **Experiment 1** | CCR | Inbreeding 1 | M | **-0.125** | **0.016** | **70** |
|  |  | Inbreeding 2 | M | -0.126 | 0.291 | 72 |
|  |  | UC | M | -0.179 | 0.250 | 43 |
|  |  | Inbreeding 1 | F | -0.208 | 0.077 | 73 |
|  |  | Inbreeding 2 | F | -0.156 | 0.170 | 79 |
|  |  | UC | F | **-0.289** | **0.049** | **47** |
|  | HKD | Inbreeding 1 | M | -0.110 | 0.407 | 59 |
|  |  | Inbreeding 2 | M | 0.071 | 0.608 | 54 |
|  |  | UC | M | 0.241 | 0.293 | 21 |
|  |  | Inbreeding 1 | F | 0.135 | 0.275 | 67 |
|  |  | Inbreeding 2 | F | **0.415** | **0.001** | **58** |
|  |  | UC | F | **0.542** | **0.002** | **30** |
| **Experiment 2** | CCR | Sel 0 Inb 0 RT 27 AT 20 F 0 | M | **-0.304** | **0.011** | **70** |
|  |  | Sel 0 Inb 0 RT 27 AT 27 F 0 | M | -0.146 | 0.249 | 64 |
|  |  | Sel 0 Inb 1 RT 27 AT 20 F 0 | M | -0.051 | 0.693 | 63 |
|  |  | Sel 0 Inb 2 RT 27 AT 20 F 0 | M | -0.088 | 0.481 | 66 |
|  |  | Sel 0 Inb 2 RT 27 AT 27 F 0 | M | -0.024 | 0.837 | 74 |
|  |  | Sel 1 Inb 0 RT 27 AT 20 F 0 | M | -0.209 | 0.129 | 54 |
|  |  | Sel 1 Inb 0 RT 27 AT 27 F 0 | M | -0.127 | 0.320 | 63 |
|  |  | Sel 1 Inb 1 RT 27 AT 20 F 0 | M | -0.178 | 0.153 | 66 |
|  |  | Sel 1 Inb 1 RT 27 AT 27 F 0 | M | **-0.359** | **0.002** | **74** |
|  |  | Sel 1 Inb 2 RT 27 AT 20 F 0 | M | -0.217 | 0.098 | 59 |
|  |  | Sel 1 Inb 2 RT 27 AT 27 F 0 | M | -0.099 | 0.452 | 60 |
|  |  | Sel 0 Inb 0 RT 20 AT 20 F 1 | M | 0.093 | 0.435 | 72 |
|  |  | Sel 0 Inb 0 RT 20 AT 27 F 1 | M | -0.078 | 0.510 | 73 |
|  |  | Sel 0 Inb 0 RT 27 AT 20 F 1 | M | 0.090 | 0.468 | 67 |
|  |  | Sel 0 Inb 0 RT 27 AT 27 F 1 | M | -0.186 | 0.092 | 83 |
|  |  | Sel 0 Inb 1 RT 20 AT 20 F 1 | M | 0.225 | 0.056 | 73 |
|  |  | Sel 0 Inb 1 RT 20 AT 27 F 1 | M | 0.055 | 0.645 | 73 |
|  |  | Sel 0 Inb 1 RT 27 AT 20 F 1 | M | **-0.328** | **0.008** | **64** |
|  |  | Sel 0 Inb 1 RT 27 AT 27 F 1 | M | -0.160 | 0.197 | 67 |
|  |  | Sel 0 Inb 2 RT 20 AT 20 F 1 | M | 0.087 | 0.473 | 71 |
|  |  | Sel 0 Inb 2 RT 20 AT 27 F 1 | M | -0.043 | 0.719 | 74 |
|  |  | Sel 0 Inb 2 RT 27 AT 20 F 1 | M | 0.033 | 0.801 | 62 |
|  |  | Sel 0 Inb 2 RT 27 AT 27 F 1 | M | 0.209 | 0.084 | 69 |
|  |  | Sel 1 Inb 0 RT 20 AT 20 F 1 | M | -0.163 | 0.177 | 70 |
|  |  | Sel 1 Inb 0 RT 20 AT 27 F 1 | M | -0.194 | 0.099 | 73 |
|  |  | Sel 1 Inb 0 RT 27 AT 20 F 1 | M | 0.030 | 0.808 | 66 |
|  |  | Sel 1 Inb 0 RT 27 AT 27 F 1 | M | 0.120 | 0.345 | 64 |
|  |  | Sel 1 Inb 1 RT 20 AT 20 F 1 | M | **0.298** | **0.010** | **74** |
|  |  | Sel 1 Inb 1 RT 20 AT 27 F 1 | M | -0.144 | 0.214 | 76 |
|  |  | Sel 1 Inb 1 RT 27 AT 20 F 1 | M | 0.065 | 0.597 | 68 |
|  |  | Sel 1 Inb 1 RT 27 AT 27 F 1 | M | 0.050 | 0.691 | 66 |
|  |  | Sel 1 Inb 2 RT 20 AT 20 F 1 | M | 0.251 | 0.055 | 59 |
|  |  | Sel 1 Inb 2 RT 20 AT 27 F 1 | M | 0.055 | 0.674 | 62 |
|  |  | Sel 1 Inb 2 RT 27 AT 20 F 1 | M | **-0.319** | **0.008** | **67** |
|  |  | Sel 1 Inb 2 RT 27 AT 27 F 1 | M | 0.204 | 0.117 | 60 |
|  |  | Sel 0 Inb 0 RT 27 AT 20 F 0 | F | 0.105 | 0.424 | 60 |
|  |  | Sel 0 Inb 0 RT 27 AT 27 F 0 | F | 0.151 | 0.243 | 62 |
|  |  | Sel 0 Inb 1 RT 27 AT 20 F 0 | F | -0.009 | 0.942 | 64 |
|  |  | Sel 0 Inb 2 RT 27 AT 20 F 0 | F | 0.030 | 0.824 | 56 |
|  |  | Sel 0 Inb 2 RT 27 AT 27 F 0 | F | 0.241 | 0.062 | 61 |
|  |  | Sel 1 Inb 0 RT 27 AT 20 F 0 | F | 0.200 | 0.116 | 63 |
|  |  | Sel 1 Inb 0 RT 27 AT 27 F 0 | F | 0.019 | 0.886 | 59 |
|  |  | Sel 1 Inb 1 RT 27 AT 20 F 0 | F | **0.308** | **0.013** | **64** |
|  |  | Sel 1 Inb 1 RT 27 AT 27 F 0 | F | 0.100 | 0.441 | 62 |
|  |  | Sel 1 Inb 2 RT 27 AT 20 F 0 | F | -0.020 | 0.870 | 70 |
|  |  | Sel 1 Inb 2 RT 27 AT 27 F 0 | F | 0.120 | 0.349 | 63 |
|  |  | Sel 0 Inb 0 RT 20 AT 20 F 1 | F | 0.090 | 0.436 | 77 |
|  |  | Sel 0 Inb 0 RT 20 AT 27 F 1 | F | 0.125 | 0.291 | 73 |
|  |  | Sel 0 Inb 0 RT 27 AT 20 F 1 | F | 0.043 | 0.728 | 68 |
|  |  | Sel 0 Inb 0 RT 27 AT 27 F 1 | F | -0.242 | 0.051 | 66 |
|  |  | Sel 0 Inb 1 RT 20 AT 20 F 1 | F | **0.322** | **0.005** | **73** |
|  |  | Sel 0 Inb 1 RT 20 AT 27 F 1 | F | -0.051 | 0.673 | 71 |
|  |  | Sel 0 Inb 1 RT 27 AT 20 F 1 | F | -0.236 | 0.075 | 58 |
|  |  | Sel 0 Inb 1 RT 27 AT 27 F 1 | F | -0.030 | 0.824 | 59 |
|  |  | Sel 0 Inb 2 RT 20 AT 20 F 1 | F | 0.043 | 0.719 | 74 |
|  |  | Sel 0 Inb 2 RT 20 AT 27 F 1 | F | -0.153 | 0.205 | 70 |
|  |  | Sel 0 Inb 2 RT 27 AT 20 F 1 | F | -0.233 | 0.079 | 58 |
|  |  | Sel 0 Inb 2 RT 27 AT 27 F 1 | F | 0.046 | 0.728 | 60 |
|  |  | Sel 1 Inb 0 RT 20 AT 20 F 1 | F | 0.024 | 0.848 | 69 |
|  |  | Sel 1 Inb 0 RT 20 AT 27 F 1 | F | 0.052 | 0.686 | 63 |
|  |  | Sel 1 Inb 0 RT 27 AT 20 F 1 | F | 0.239 | 0.055 | 65 |
|  |  | Sel 1 Inb 0 RT 27 AT 27 F 1 | F | -0.181 | 0.135 | 70 |
|  |  | Sel 1 Inb 1 RT 20 AT 20 F 1 | F | 0.204 | 0.079 | 75 |
|  |  | Sel 1 Inb 1 RT 20 AT 27 F 1 | F | -0.011 | 0.928 | 71 |
|  |  | Sel 1 Inb 1 RT 27 AT 20 F 1 | F | -0.033 | 0.793 | 67 |
|  |  | Sel 1 Inb 1 RT 27 AT 27 F 1 | F | **-0.255** | **0.041** | **65** |
|  |  | Sel 1 Inb 2 RT 20 AT 20 F 1 | F | **0.278** | **0.033** | **59** |
|  |  | Sel 1 Inb 2 RT 20 AT 27 F 1 | F | 0.068 | 0.618 | 56 |
|  |  | Sel 1 Inb 2 RT 27 AT 20 F 1 | F | -0.194 | 0.156 | 55 |
|  |  | Sel 1 Inb 2 RT 27 AT 27 F 1 | F | 0.231 | 0.060 | 67 |
