# Supplementary material for: No Trade-Off between Growth Rate and Temperature Stress Resistance in Four Insect Species
Source: PLoS One. 2013 Apr 30;8(4):e62434. doi: 10.1371/journal.pone.0062434 (PMC3640073; doi:10.1371/journal.pone.0062434)
Supplement: Table S10 — Experiments 3–7 (Lycaena tityrus). Within-group correlations between growth rate and temperature stress resistance (chill coma recovery, CCR and/or heat knock down time, HKD) for the butterfly Lycaena tityrus in experiments 3–7 (N = 8–16 correlations per trait). RT = rearing temperature (18, 19, 20, 24 or 27°C); AT = acclimation temperature (20 or 27°C); const. = const rearing conditions, fluct. = fluctuating rearing conditions; low = low-altitude populations (0–600 m), mid = mid-altitude populations (1300–1500 m), high = high-altitude populations (1900–2100 m); G1 = PGI 1–1, G2 = PGI 2–2, G3 = PGI 1–2, G4 = PGI 2–3 genotypes; M = male, F = female. Significant correlations are given in bold. (DOCX) [file pone.0062434.s010.docx]

**Table S10**

| **Exp.** | **Trait** | **Treatment group** | **Sex** | **R** | **P** | **N** |
| --- | --- | --- | --- | --- | --- | --- |
| **Experiment 3** | CCR | RT 20 AT 20 | M | -0.357 | 0.067 | 27 |
|  |  | RT 20 AT 27 | M | -0.129 | 0.539 | 25 |
|  |  | RT 27 AT 20 | M | -0.056 | 0.715 | 45 |
|  |  | RT 27 AT 27 | M | -0.030 | 0.867 | 33 |
|  |  | RT 20 AT 20 | F | 0.011 | 0.941 | 51 |
|  |  | RT 20 AT 27 | F | -0.154 | 0.377 | 35 |
|  |  | RT 27 AT 20 | F | -0.012 | 0.926 | 65 |
|  |  | RT 27 AT 27 | F | **0.412** | **0.001** | **61** |
| **Experiment 4** | CCR | RT 18 const | M | 0.206 | 0.208 | 39 |
|  |  | RT 18 fluct | M | **0.357** | **0.024** | **40** |
|  |  | RT 24 const | M | **0.413** | **0.007** | **41** |
|  |  | RT 24 fluct | M | 0.079 | 0.663 | 33 |
|  |  | RT 18 const | F | 0.201 | 0.190 | 44 |
|  |  | RT 18 fluct | F | 0.163 | 0.202 | 63 |
|  |  | RT 24 const | F | **0.459** | **0.001** | **50** |
|  |  | RT 24 fluct | F | 0.195 | 0.113 | 67 |
|  | HKD | RT 18 const | M | -0.209 | 0.202 | 39 |
|  |  | RT 18 fluct | M | -0.053 | 0.748 | 39 |
|  |  | RT 24 const | M | -0.119 | 0.472 | 39 |
|  |  | RT 24 fluct | M | **-0.346** | **0.048** | **33** |
|  |  | RT 18 const | F | 0.253 | 0.098 | 44 |
|  |  | RT 18 fluct | F | 0.103 | 0.425 | 62 |
|  |  | RT 24 const | F | -0.174 | 0.238 | 48 |
|  |  | RT 24 fluct | F | -0.161 | 0.196 | 66 |
| **Experiment 5** | CCR | RT 18 low | M | **-0.318** | **0.040** | **42** |
|  |  | RT 27 low | M | 0.156 | 0.152 | 86 |
|  |  | RT 18 high | M | 0.008 | 0.967 | 31 |
|  |  | RT 27 high | M | -0.084 | 0.519 | 61 |
|  |  | RT 18 low | F | 0.037 | 0.866 | 23 |
|  |  | RT 27 low | F | 0.101 | 0.390 | 74 |
|  |  | RT 18 high | F | 0.312 | 0.181 | 20 |
|  |  | RT 27 high | F | 0.069 | 0.575 | 68 |
|  | HKD | RT 18 low | M | -0.101 | 0.526 | 42 |
|  |  | RT 27 low | M | 0.180 | 0.104 | 83 |
|  |  | RT 18 high | M | **-0.403** | **0.025** | **31** |
|  |  | RT 27 high | M | 0.227 | 0.078 | 61 |
|  |  | RT 18 low | F | 0.161 | 0.475 | 22 |
|  |  | RT 27 low | F | 0.163 | 0.166 | 74 |
|  |  | RT 18 high | F | -0.260 | 0.268 | 20 |
|  |  | RT 27 high | F | -0.100 | 0.419 | 68 |
| **Experiment 6** | CCR | low | M | **-0.312** | **0.014** | **61** |
|  |  | mid | M | 0.043 | 0.784 | 43 |
|  |  | high | M | 0.144 | 0.325 | 49 |
|  |  | low | F | -0.143 | 0.368 | 42 |
|  |  | mid | F | 0.229 | 0.150 | 41 |
|  |  | high | F | -0.115 | 0.451 | 45 |
|  | HKD | low | M | -0.033 | 0.785 | 72 |
|  |  | mid | M | 0.096 | 0.481 | 56 |
|  |  | high | M | 0.294 | 0.108 | 31 |
|  |  | low | F | **0.312** | **0.035** | **46** |
|  |  | mid | F | **-0.371** | **0.020** | **39** |
|  |  | high | F | 0.155 | 0.360 | 37 |
| **Experiment 7** | CCR | RT 19 G1 | M | -0.288 | 0.341 | 13 |
|  |  | RT 19 G2 | M | -0.147 | 0.279 | 56 |
|  |  | RT 19 G3 | M | -0.201 | 0.093 | 71 |
|  |  | RT 19 G4 | M | 0.063 | 0.780 | 22 |
|  |  | RT 24 G1 | M | -0.517 | 0.126 | 10 |
|  |  | RT 24 G2 | M | 0.043 | 0.725 | 69 |
|  |  | RT 24 G3 | M | 0.000 | 0.999 | 79 |
|  |  | RT 24 G4 | M | 0.345 | 0.208 | 15 |
|  |  | RT 19 G1 | F | 0.007 | 0.979 | 17 |
|  |  | RT 19 G2 | F | 0.220 | 0.124 | 50 |
|  |  | RT 19 G3 | F | 0.021 | 0.877 | 59 |
|  |  | RT 19 G4 | F | 0.182 | 0.500 | 16 |
|  |  | RT 24 G1 | F | 0.361 | 0.379 | 8 |
|  |  | RT 24 G2 | F | 0.180 | 0.165 | 61 |
|  |  | RT 24 G3 | F | 0.052 | 0.621 | 94 |
|  |  | RT 24 G4 | F | -0.088 | 0.809 | 10 |
|  | HKD | RT 19 G1 | M | 0.275 | 0.387 | 12 |
|  |  | RT 19 G2 | M | **0.355** | **0.005** | **61** |
|  |  | RT 19 G3 | M | 0.239 | 0.054 | 66 |
|  |  | RT 19 G4 | M | -0.076 | 0.778 | 16 |
|  |  | RT 24 G1 | M | -0.281 | 0.589 | 6 |
|  |  | RT 24 G2 | M | 0.172 | 0.135 | 77 |
|  |  | RT 24 G3 | M | 0.068 | 0.531 | 88 |
|  |  | RT 24 G4 | M | 0.413 | 0.309 | 8 |
|  |  | RT 19 G1 | F | 0.307 | 0.359 | 11 |
|  |  | RT 19 G2 | F | 0.110 | 0.455 | 48 |
|  |  | RT 19 G3 | F | **0.466** | **0.000** | **64** |
|  |  | RT 19 G4 | F | 0.137 | 0.554 | 21 |
|  |  | RT 24 G1 | F | 0.436 | 0.241 | 9 |
|  |  | RT 24 G2 | F | -0.013 | 0.919 | 64 |
|  |  | RT 24 G3 | F | **0.354** | **0.001** | **84** |
|  |  | RT 24 G4 | F | -0.028 | 0.908 | 19 |
