# Supplementary material for: No Trade-Off between Growth Rate and Temperature Stress Resistance in Four Insect Species
Source: PLoS One. 2013 Apr 30;8(4):e62434. doi: 10.1371/journal.pone.0062434 (PMC3640073; doi:10.1371/journal.pone.0062434)
Supplement: Table S11 — Experiments 8–10 (Pieris napi). Within-group correlations between growth rate and temperature stress resistance (chill-coma recovery, CCR and/or heat knock-down time, HKD) for the butterfly Pieris napi in experiments 8–10 (N = 4–8 correlations per trait). TR1 = mean temperature: 17°C, amplitude 7°C; TR2 = mean temperature: 20°C, amplitude: 7°C; TR3 = mean temperature: 20°C, amplitude: 12°C; TR 4 = mean temperature of 17°C and amplitude of 7°C during the first half of larval development and mean temperature of 27°C and amplitude of 7°C during the second half of larval development; RT = rearing temperature (19, 20, 25 or 27°C); M = male, F = female. Significant correlations are given in bold. (DOCX) [file pone.0062434.s011.docx]

**Table S11**

| **Exp.** | **Trait** | **Treatment group** | **Sex** | **R** | **P** | **N** |
| --- | --- | --- | --- | --- | --- | --- |
| **Experiment 8** | CCR | T1 | M | **0.342** | **0.041** | **36** |
|  |  | T2 | M | -0.206 | 0.243 | 34 |
|  |  | T3 | M | 0.088 | 0.590 | 40 |
|  |  | T4 | M | -0.165 | 0.487 | 20 |
|  |  | T1 | F | 0.104 | 0.527 | 39 |
|  |  | T2 | F | -0.315 | 0.070 | 34 |
|  |  | T3 | F | -0.142 | 0.403 | 37 |
|  |  | T4 | F | -0.232 | 0.388 | 16 |
|  | HKD | T1 | M | 0.225 | 0.215 | 32 |
|  |  | T2 | M | 0.188 | 0.321 | 30 |
|  |  | T3 | M | -0.224 | 0.196 | 35 |
|  |  | T4 | M | -0.383 | 0.106 | 19 |
|  |  | T1 | F | 0.102 | 0.544 | 38 |
|  |  | T2 | F | 0.073 | 0.706 | 29 |
|  |  | T3 | F | 0.130 | 0.457 | 35 |
|  |  | T4 | F | -0.506 | 0.078 | 13 |
| **Experiment 9** | CCR | RT 20 | M | 0.049 | 0.693 | 65 |
|  |  | RT 27 | M | -0.372 | 0.056 | 27 |
|  |  | RT 20 | F | -0.102 | 0.440 | 60 |
|  |  | RT 27 | F | -0.374 | 0.072 | 24 |
|  | HKD | RT 20 | M | 0.175 | 0.164 | 65 |
|  |  | RT 27 | M | -0.093 | 0.646 | 27 |
|  |  | RT 20 | F | **-0.289** | **0.025** | **60** |
|  |  | RT 27 | F | -0.076 | 0.724 | 24 |
| **Experiment 10** | CCR | RT 19 | M | 0.106 | 0.343 | 82 |
|  |  | RT 25 | M | -0.013 | 0.892 | 105 |
|  |  | RT 19 | F | -0.112 | 0.341 | 75 |
|  |  | RT 25 | F | **-0.183** | **0.008** | **100** |
|  | HKD | RT 19 | M | 0.071 | 0.531 | 80 |
|  |  | RT 25 | M | -0.111 | 0.273 | 100 |
|  |  | RT 19 | F | -0.100 | 0.375 | 80 |
|  |  | RT 25 | F | 0.144 | 0.164 | 95 |
