# Supplementary material for: No Trade-Off between Growth Rate and Temperature Stress Resistance in Four Insect Species
Source: PLoS One. 2013 Apr 30;8(4):e62434. doi: 10.1371/journal.pone.0062434 (PMC3640073; doi:10.1371/journal.pone.0062434)
Supplement: Table S12 — Experiment 11 (Protophormia terraenovae). Within-group correlations between growth rate and chill-coma recovery (CCR) for the fly Protophormia terranovae in experiment 11 (N = 8 correlations). RT = rearing temperature (20 or 27°C); 12 h/18 h = photoperiod of 12 or 18 h; M = male, F = female. Significant correlations are given in bold. (DOCX) [file pone.0062434.s012.docx]

**Table S12**

| **Exp.** | **Trait** | **Treatment group** | **Sex** | **R** | **P** | **N** |
| --- | --- | --- | --- | --- | --- | --- |
| **Experiment 11** | CCR | RT 20 12h | M | 0.343 | 0.094 | 25 |
|  |  | RT 20 18h | M | 0.435 | 0.081 | 17 |
|  |  | RT 27 12h | M | 0.042 | 0.832 | 28 |
|  |  | RT 27 18h | M | -0.250 | 0.191 | 29 |
|  |  | RT 20 12h | F | -0.197 | 0.391 | 21 |
|  |  | RT 20 18h | F | -0.285 | 0.127 | 30 |
|  |  | RT 27 12h | F | **0.454** | **0.044** | **20** |
|  |  | RT 27 18h | F | -0.282 | 0.257 | 18 |
